# Comparative Effectiveness of Exercise, Protein Supplementation, and Combined Interventions for Sarcopenia Management in Women: A Network Meta-Analysis

**DOI:** 10.3390/nu17152392

**Published:** 2025-07-22

**Authors:** Ruixiang Yan, Wenrui Huang, Yuanhao Zhong, Xuelian Du

**Affiliations:** 1 School of Athletic Training, Guangzhou Sport University, Guangzhou 510500, China; 2 The Fourth Clinical Medical College, Guangzhou University of Chinese Medicine, Guangzhou 510405, China; 3 College of Physical Education, Beijing Sport University, Beijing 100084, China

**Keywords:** sarcopenia, women, exercise, protein supplementation

## Abstract

**Background/Objectives**: The comparative efficacy and optimal combination strategies of exercise intervention, nutritional supplementation, and their integration for older women with sarcopenia remain inadequately supported by high-quality evidence. **Methods**: We systematically searched PubMed, Embase, Web of Science, and the Cochrane Central Register of Controlled Trials (CENTRAL) until February 2025. A frequentist random-effects network meta-analysis was conducted to compare the relative effects of different interventions. The quality of evidence was assessed using the GRADE framework, and interventions were ranked based on relative efficacy and evidence certainty. **Results**: A total of 21 randomized controlled trials involving 1215 participants were included. The network meta-analysis showed that combined exercise and nutritional interventions were the most effective in improving handgrip strength (MD = 1.95, 95% CI: 0.1 to 3.18; SUCRA = 74%), usual gait speed (MD = 0.11, 95% CI: 0.04 to 0.17; SUCRA = 94.49%), maximum gait speed (MD = 0.22, 95% CI: 0.06 to 0.38; SUCRA = 82.17%), and appendicular skeletal muscle mass (MD = 0.21, 95% CI: 0.05 to 0.38; SUCRA = 92.83%). Exercise alone significantly improved knee extension strength (SMD = 0.75, 95% CI: 0.41 to 1.08; SUCRA = 84.58%). However, nutritional supplementation alone did not significantly improve any outcome. No intervention demonstrated a significant effect on skeletal muscle mass index. **Conclusion:** Exercise interventions effectively enhance muscle mass, strength, and physical function in older women with sarcopenia. Combined exercise and nutritional supplementation may offer superior benefits compared with exercise alone.

## 1. Introduction

Sarcopenia is an age-related progressive skeletal muscle disorder primarily characterized by a reduction in skeletal muscle mass, decreased muscle strength, and impaired physical function [1,2]. Numerous studies have shown that sarcopenia is closely associated with a range of adverse health outcomes, including falls, physical disability, functional decline, prolonged hospitalization, frailty, and even death [1,2]. A systematic review and meta-analysis reported that the prevalence of sarcopenia ranges from 10% to 27%, with severe sarcopenia affecting approximately 2% to 9% of individuals [3]. With the ongoing global aging of the population, the prevalence of sarcopenia is expected to rise further, leading to increasing demands on healthcare systems and escalating economic burdens [4].

The management of sarcopenia has emerged as a key issue in geriatric medicine, yet consensus on the optimal intervention strategy has not been fully established. In current clinical practice, resistance training (RT) is widely regarded as the most evidence-based approach for treating sarcopenia, demonstrating significant improvements in muscle mass, strength, and physical function and earning strong recommendations supported by high-certainty evidence [5,6]. However, for most frail or functionally limited middle-aged and older adults, performing high-intensity or complex multi-joint resistance training is often challenging [7,8,9]. As such, relying solely on conventional resistance training to preserve or enhance physical function may have limited applicability and cost-effectiveness in this population [9]. In contrast, multi-modal exercise interventions incorporating aerobic or balance training alongside resistance training may offer more comprehensive improvements in functional status [10,11]. In addition, protein supplementation has been conditionally recommended as an adjunct strategy, particularly when combined with exercise, which shows greater benefits [12,13]. In comparison, current evidence regarding vitamin D [14], anabolic hormones [15], and pharmacological therapies [16,17] remains insufficient to support their recommendation as first-line interventions.

A recent nationwide cohort study identified female sex as an independent risk factor for sarcopenia [18]. Compared with men, older women exhibit a higher prevalence of sarcopenia and an increased risk of functional decline, which may be attributed to several factors: lower innate muscle reserves [19,20], diminished anabolic protection due to estrogen deficiency [21,22], and elevated levels of pro-inflammatory cytokines such as interleukin-6 [23]. These factors collectively contribute to significantly increased risks of falls, fractures, and all-cause mortality in older women [24]. Given the well-established physiological differences between older men and women, several studies have specifically investigated the effectiveness of exercise and nutritional interventions in older women. A meta-regression analysis indicated that a higher proportion of female participants was significantly associated with greater improvements in walking speed and more limited gains in skeletal muscle mass index following exercise interventions, suggesting that sex may be an important source of heterogeneity in exercise efficacy [25]. Two recent meta-analyses have preliminarily explored the effects of exercise training (including resistance and aerobic exercise) and vitamin D supplementation in preventing sarcopenia among healthy middle-aged women [26,27]. These studies confirmed that such interventions could significantly delay a decline in muscle mass and strength; however, the evidence for improvements in physical function remains insufficient.

Notably, most of these studies were conducted in general populations without a confirmed diagnosis of sarcopenia, making it difficult to generalize the findings to high-risk or clinically diagnosed individuals. To address this gap, the present study conducted a network meta-analysis integrating direct and indirect evidence to systematically compare the effects of exercise intervention, protein-based nutritional supplementation, and their combination on core outcomes among middle-aged and older women with sarcopenia. All interventions were ranked using a minimally contextualized framework. In addition, subgroup analyses based on different exercise modalities were conducted to provide evidence-based guidance for developing more targeted clinical intervention strategies.

## 2. Methods

### 2.1. Protocol and Registration

The systematic review and network meta-analysis were prospectively registered in PROSPERO (Registration No.: CRD420251066238). The study adhered to the Preferred Reporting Items for Systematic Reviews and Meta-Analyses (PRISMA) 2020 guidelines and its extension statement for network meta-analyses (PRISMA-NMA) [28,29].

### 2.2. Search Strategy and Study Selection

A systematic literature search was conducted in PubMed, Web of Science, Cochrane CENTRAL, and Embase databases for randomized controlled trials (RCTs) investigating the effects of different exercise modalities and nutritional interventions in middle-aged and older women with sarcopenia. The search included studies published up to 16 February 2025. Three reviewers (GS, BW, and LX) independently performed the search and screened eligible studies. Disagreements were resolved through discussion with a fourth reviewer (YE). Additionally, we screened the reference lists of included studies and relevant systematic reviews to identify potential eligible trials. The whole search strategy is presented in Section S1.

### 2.3. Eligibility Criteria

Eligibility was assessed using the PICOS framework (Population, Intervention, Comparator, Outcome, and Study Design) [30]. Studies meeting all of the following criteria were included.

#### 2.3.1. Population

We included studies enrolling middle-aged and older women (>50 years) diagnosed with or at risk of sarcopenia. We did not impose a unified diagnostic criterion. However, we accepted the definitions used in the original studies, including (but not limited to) those proposed by authoritative bodies such as the EWGSOP or AWGS, or other study-defined criteria. All accepted definitions had to include at least one of the three core domains: low muscle mass, low muscle strength, or impaired physical function.

#### 2.3.2. Intervention

We included interventions involving any exercise and nutritional supplementation primarily based on protein.

#### 2.3.3. Comparator

Control groups included any of the above interventions, such as health education, routine care, or placebo.

#### 2.3.4. Outcome

The outcome measures included muscle strength, muscle mass, and physical function, detailed as follows: Muscle strength: Handgrip strength, knee extension strength; Physical function. Usual gait speed, maximal gait speed; Muscle mass: Skeletal muscle mass index (SMI), appendicular skeletal muscle mass (ASM).

#### 2.3.5. Study Design

Only RCTs were included.The exclusion criteria were as follows: (1) participants diagnosed with sarcopenia secondary to specific conditions (e.g., cancer, diabetes, stroke, HIV, chronic obstructive pulmonary disease, chronic kidney disease, liver cirrhosis, other serious illnesses, or recent organ transplantation); (2) interventions involving pharmacological treatments; (3) conference abstracts, study protocols, or systematic reviews; (4) non-English publications; (5) studies with insufficient outcome data; and (6) studies for which complete reports could not be retrieved from any accessible source.

### 2.4. Data Extraction

Two reviewers (GS, BW) independently extracted data for each eligible study using a predesigned extraction form. Extracted data included study characteristics (first author, publication year, country, sarcopenia diagnostic criteria), participant characteristics (age, sample size), intervention characteristics (type of intervention, duration, nutritional dosage), and outcome data (means and standard deviations for continuous outcomes, proportions or event rates for binary outcomes). Data extraction was verified by a third reviewer (LX). In cases where data were missing, we attempted to contact the corresponding author thrice within 3 weeks.

### 2.5. Measures of Treatment Effect

We used mean differences (MD) and standard deviations (SD) to assess treatment effects. If SD were not directly reported, they were estimated from standard errors, 95% confidence intervals (CI), *p*-values, or t-statistics [31]. Where pre–post change SD were unavailable, they were estimated using the following formula:

$${SD}_{change}=\sqrt{{SD}_{baseline}^{2}+{SD}_{Post}^{2}-2\times r\times {SD}_{baseline}\times {SD}_{post}}$$

This formula assumed a correlation coefficient (r) of 0.5, reflecting moderate measurement reproducibility, which is widely accepted in the prior literature. This value was chosen to balance potential variability and ensure the robustness and reliability of effect estimates [31].

### 2.6. Quality Assessment of Evidence

The risk of bias in included RCTs was assessed using the Cochrane Risk of Bias 2.0 (ROB 2.0) tool, covering domains like random sequence generation, allocation concealment, blinding, missing outcome data, and selective outcome reporting [32]. Each study was classified as follows: low risk of bias (score = 1) if all domains were rated low risk, high risk of bias (score = 3) if at least one domain was rated high risk, and some concerns (score = 2) in all other cases. Two reviewers conducted assessments independently, and disagreements were resolved through consensus.

To assess small-study effects and publication bias, funnel plots were constructed for each direct comparison. The certainty of the evidence was further evaluated using the CINeMA framework, covering six key domains: within-study bias, reporting bias, indirectness, imprecision, heterogeneity, and incoherence [33,34]. Each domain assessed the presence of systematic errors, selective reporting, relevance of evidence to the research question, range of effect estimate uncertainty, consistency of results, and agreement between direct and indirect comparisons.

### 2.7. Minimally Contextualized Framework

A minimally contextualized framework was adopted to assess imprecision and classify intervention effects relative to the control group [35]. The null value (MD = 0) was used as the threshold to categorize interventions as follows [36,37]: Among the most effective, the point estimate notably favors the intervention, and the 95% CI excludes the null. For intermediately effective, the point estimate favors the intervention, but the 95% CI includes or approaches the null. Among the least effective, the point estimate is close to the null, and the 95% CI includes the null. In addition, the certainty of the evidence was categorized into two levels, high/moderate or low/very low, based on the GRADE framework to aid the interpretation of intervention credibility [35]. To determine the clinical relevance of intervention effects, we referred to established minimal important difference (MID) thresholds for sarcopenia-related outcomes. For handgrip strength, we applied an MID of 5 kg, originally proposed by Bohannon et al. [38] in older and function-limited populations and now the benchmark most often cited in sarcopenia research [11]. A later systematic review with a meta-analysis by Bobos et al. [39] showed that MID estimates vary (≈2.7 kg in healthy older adults to 6.5 kg in post-fracture rehabilitation); these data nonetheless support 5 kg as a conservative, clinically meaningful threshold for frail older people. For usual gait speed, a consensus MID of 0.10 m/s was applied [40].

### 2.8. Statistical Analysis

A frequentist network meta-analysis was performed using the netmeta package in R (version 4.3.1), based on a graph-theoretical approach. Effect estimates were derived using weighted least squares regression with the Moore–Penrose generalized inverse. A random-effects model was applied to account for between-study heterogeneity [41,42]. For outcomes reported using the same scale or units, MD was used. For outcomes measured using different scales or instruments (e.g., knee extension strength, ASM), Standardized Mean Difference (SMD) and 95% CI were calculated to ensure comparability.

Heterogeneity was assessed using the generalized Cochran’s Q statistic to evaluate global and local inconsistency within the network. The node-splitting method examined agreement between direct and indirect evidence; *p*-values < 0.05 indicated statistically significant inconsistency [43]. The structure of the treatment network was visualized using network plots, with nodes representing interventions and edges representing direct comparisons. To illustrate effect estimates, we generated forest plots and league tables comparing relative effects across interventions. Surface under the cumulative ranking curve (SUCRA) values were calculated to rank interventions and visualized using rank heat plots generated via the online platform RankHeatPlot (available at https://rankheatplot.com/rankheatplot/, accessed on 25 May 2025) [44,45]. Publication bias was further evaluated using funnel plots, which was confirmed using Egger’s test.

Subgroup analyses were conducted to explore the potential influence of exercise modality, with exercise type as the primary grouping variable and the constant nutritional intervention. Interventions were classified into distinct categories: different types of exercise alone, different types of exercise combined with nutritional supplementation, and nutritional supplementation alone.

## 3. Results

### 3.1. Literature Selection and Study Characteristics

**Figure 1:**
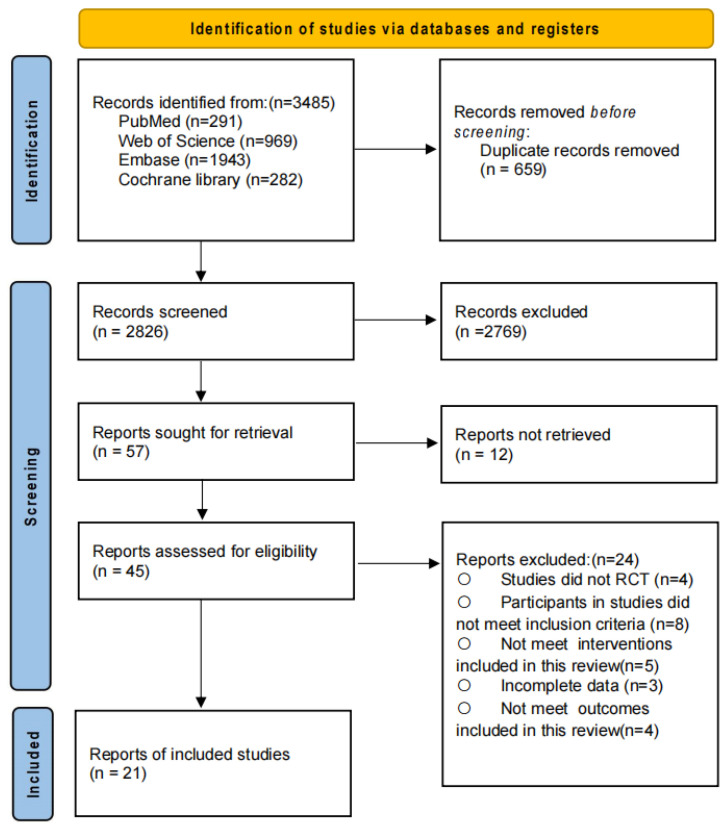
PRISMA flow diagram of the search process for studies.

Through systematic searching, 3485 potentially relevant records were identified. After removing duplicates, 2826 articles remained for title and abstract screening. Of these, 57 articles met the criteria for full-text review. Ultimately, 21 studies were included in this review and meta-analysis, involving 1215 participants with a mean age of 71.1  ±  7.66 years. The complete screening and selection process is illustrated in Figure 1. Characteristics of the included studies are summarized in Table 1, and the detailed search strategy is provided in Section S1.

### 3.2. Risk of Bias, Certainty of Evidence, and Consistency

**Figure 2:**
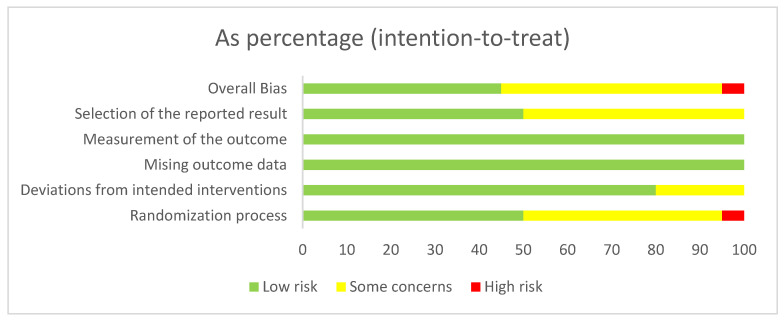
Summary of the risk of bias assessment in the individual domains of the included studies.

Overall, 9 studies (45%) were rated as low risk of bias, 11 studies (50%) were rated as having some concerns, and 1 study (5%) was rated as high risk of bias (Figure 2). Risk of bias assessments for individual studies are detailed in Section S2. We used the design-by-treatment interaction model for global inconsistency and the node-splitting method for local inconsistency to assess consistency. Neither method revealed statistically significant inconsistency (*p* > 0.05). According to the CINeMA framework, the certainty of evidence was mainly low to very low across pairwise comparisons (Section S6). Rankings based on the minimally contextualized framework are shown in Table 2. Funnel plot analysis revealed no signs of asymmetry, and Egger’s test was non-significant, indicating no clear evidence of publication bias (Section S7).

### 3.3. Muscle Strength

**Figure 3:**
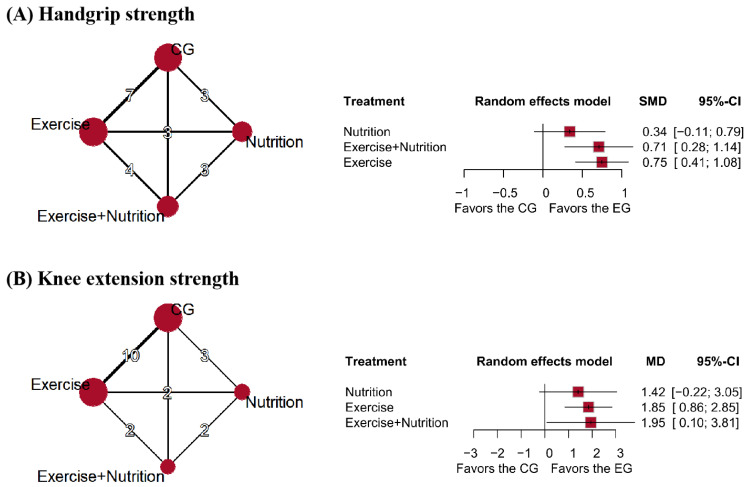
Network plots and forest plots of interventions for (**A**) grip strength, (**B**) knee extension strength.

Eleven studies (*n* = 641) reported changes in handgrip strength (Figure 3). Low certainty evidence indicated that exercise + nutrition (MD = 1.95, 95% CI: 0.10 to 3.18; SUCRA = 74%) and exercise alone (MD = 1.85, 95% CI: 0.86 to 2.85; SUCRA = 71.8%) were the most effective interventions for improving grip strength. However, the confidence intervals did not reach the predefined MID threshold of 5 kg [38,39]. Nutrition alone did not show significant effects (MD = 1.42, 95% CI: −0.22 to 3.05; SUCRA = 52.05%) (Figure 3A).

Eight studies (*n* = 636) reported changes in knee extension strength. Low certainty evidence showed that exercise (SMD = 0.75, 95% CI: 0.41 to 1.08; SUCRA = 84.58%) and exercise + nutrition (SMD = 0.71, 95% CI: 0.28 to 1.14; SUCRA = 78.81%) significantly improved knee extensor strength, and they were classified as the most effective interventions. Nutrition alone showed no significant effect (SMD = 0.34, 95% CI: −0.11 to 0.79; SUCRA = 34.3%) and was classified as relatively ineffective (Figure 3B).

### 3.4. Physical Function

**Figure 4:**
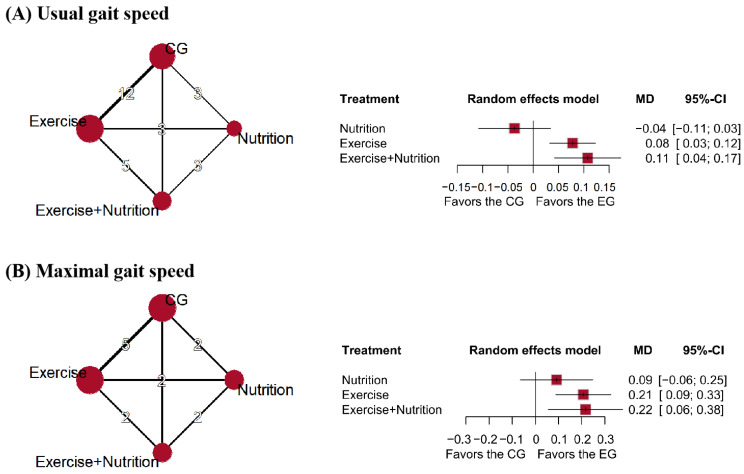
Network plots and forest plots of interventions for (**A**) usual gait speed, (**B**) maximal gait speed.

Fourteen studies (*n* = 881) reported changes in usual gait speed (Figure 4). Low certainty evidence indicated that exercise + nutrition (MD = 0.11, 95% CI: 0.04 to 0.17; SUCRA = 94.49%) and exercise (MD = 0.08, 95% CI: 0.03 to 0.12; SUCRA = 72.17%) were the most effective interventions, both exceeding the predefined MID threshold of 0.10 m/s [40]. Nutrition alone showed no apparent effect (MD = −0.04, 95% CI: −0.11 to 0.03; SUCRA = 5.11%) (Figure 4A).

Five studies (*n* = 433) reported changes in maximal gait speed. Low certainty evidence showed that exercise + nutrition (MD = 0.22, 95% CI: 0.06 to 0.38; SUCRA = 82.17%) and exercise (MD = 0.21, 95% CI: 0.09 to 0.33; SUCRA = 79.37%) were the most effective interventions, while nutrition showed no significant effect (MD = 0.09, 95% CI: −0.06 to 0.25; SUCRA = 34.12%) (Figure 4B).

### 3.5. Muscle Mass

**Figure 5:**
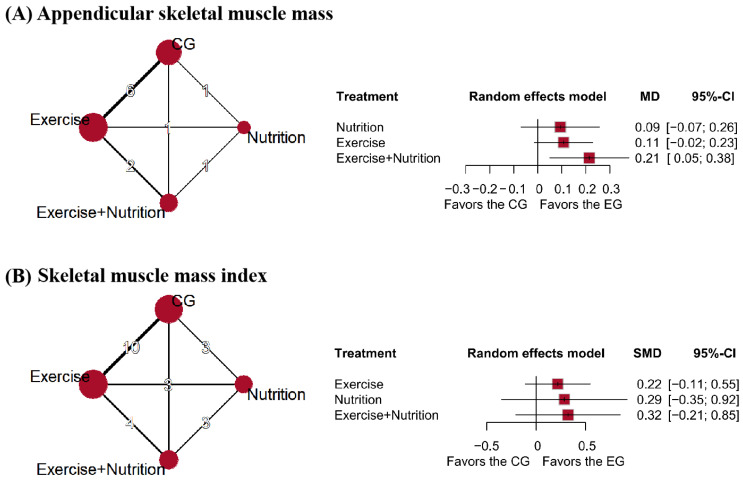
Network plot and forest plot of interventions for (**A**) appendicular skeletal muscle mass, (**B**) skeletal muscle mass index.

Ten studies (*n* = 636) reported changes in ASM. High certainty evidence showed that exercise + nutrition (MD = 0.21 kg, 95% CI: 0.05 to 0.38; SUCRA = 92.83%) was the most effective intervention. Low certainty evidence suggested that exercise alone (MD = 0.11 kg, 95% CI: −0.02 to 0.23; SUCRA = 54.04%) was moderately effective. Nutrition alone (MD = 0.09 kg, 95% CI: −0.07 to 0.26; SUCRA = 47.14%) lacked sufficient evidence to support effectiveness (Figure 5A).

**Figure 6:**
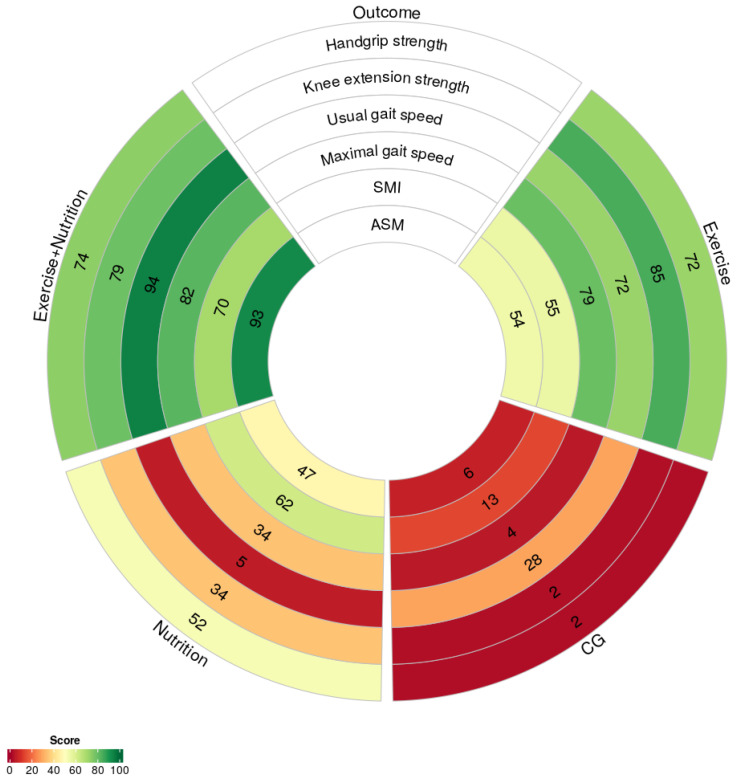
Rank heat plot of intervention effectiveness in relation to sarcopenia-related outcomes. SMI = skeletal muscle mass index; ASM = appendicular skeletal muscle mass.

Seven studies (*n* = 419) reported changes in skeletal muscle mass index (SMI). Low certainty evidence showed that exercise + nutrition (SMD = 0.32, 95% CI: −0.21 to 0.85; SUCRA = 69.75%), nutrition (SMD = 0.29, 95% CI: −0.35 to 0.92; SUCRA = 61.71%), and exercise (SMD = 0.22, 95% CI: −0.11 to 0.55; SUCRA = 55.2%) all failed to demonstrate statistically significant improvements and were thus classified as relatively ineffective interventions (Figure 5B). The SUCRA-based intervention rankings are presented in Figure 6.

### 3.6. Subgroup Analysis

To evaluate the differential effects of various exercise modalities in middle-aged and older women with sarcopenia, we conducted subgroup analyses based on exercise type. The results indicated that resistance training and nutrition (RT + Nu) improved several key outcomes, including usual gait speed, knee extension strength, and ASM. RT showed the most significant effect on handgrip strength, while resistance balance training (RBT) appeared to be the most effective for improving maximal gait speed (Section S8).

### 3.7. Sensitivity Analysis

To assess the robustness of the network meta-analysis findings, three sensitivity analyses were conducted by excluding (1) studies with a high risk of bias, (2) studies with fewer than 15 participants per group, and (3) studies including participants with possible (but not confirmed) sarcopenia. All sensitivity analyses yielded results consistent with the primary analysis, with minimal changes in effect estimates and no substantial differences in direction or magnitude, thereby supporting the robustness of our findings (Section S9).

## 4. Discussion

### 4.1. Main Findings

This review included 21 randomized controlled trials with a total of 1215 participants. To our knowledge, previous meta-analyses investigating exercise and nutrition interventions have predominantly used mixed-gender samples, and few have focused specifically on women with sarcopenia. Therefore, this study represents the first network meta-analysis to systematically compare the relative efficacy of exercise, protein-based nutritional supplementation, and their combination in this population. It provides important evidence to inform targeted interventions for women with sarcopenia. The primary findings indicate that combining exercise and protein supplementation is the most effective strategy for improving muscle strength, muscle mass, and physical function.

In contrast, protein supplementation alone failed to improve any outcomes significantly and, in some cases, was associated with further declines in physical function. We conducted a prespecified subgroup analysis to further explore the relative effects of different exercise modalities when combined with nutrition. The results revealed that resistance training and protein supplementation conferred clear advantages in enhancing knee extension strength, usual gait speed, and ASM. Additionally, multi-component training approaches such as RBT and aerobic resistance training (ART), improved physical function.

### 4.2. Comparison with Other Studies

Previous meta-analyses have found that exercise [67,68] and exercise combined with nutrition [68,69] can significantly improve grip strength, knee extensor strength, gait speed, and muscle mass, which is consistent with our findings. Luo et al. [70] demonstrated that protein supplementation may augment the effects of exercise in older adults with sarcopenia, with greater improvements in lean mass and muscle mass, as well as in knee extension strength and usual gait speed. Similarly, Liao [71] and Cuyul-Vásquez et al. [72] found that resistance training plus protein supplementation significantly outperformed resistance training alone in enhancing lean mass and leg strength. However, subgroup analyses by Liao et al. [71] indicated that such combined benefits were more prominent in men, and no significant differences in lean mass or leg strength were observed in women when comparing combined versus exercise-only interventions.

Whether protein supplementation alone can improve physical function remains controversial. While some studies suggest benefits [73,74], others report limited effects [11,68,75,76]. Notably, several meta-analyses have consistently shown that protein supplementation improves muscle strength, regardless of its effects on function [11,68,76,77]. In contrast, our study did not observe any benefit from protein supplementation alone for key outcomes in middle-aged and older women with sarcopenia. Sex-specific physiological differences may explain this discrepancy. Hormonal changes during menopause can impair protein metabolism and reduce anabolic efficiency [78]. Moreover, inadequate protein intake is more common among women over 50 (9–24%) compared with men (1–5%), placing older women at a higher risk of malnutrition [79]. A cross-sectional study also found that older women with sarcopenia were more likely than men to have impaired physical function (48.5% vs. 36.0%), limitations in basic activities of daily living (37.0% vs. 24.4%), and reduced instrumental activities of daily living (25.6% vs. 17.8%) [80]. These data suggest that older women with sarcopenia may require higher protein intake to support functional improvements, particularly at doses ≥1.11 g/kg/day [80]. Although sex-based differences in protein metabolism exist, postmenopausal women may still benefit from protein supplementation if combined with resistance training [81]. Our subgroup analyses support this view, showing that resistance training with protein supplementation was superior to other interventions in improving usual gait speed. Furthermore, we observed that multi-modal interventions, such as RBT and ART, produced favorable outcomes in usual gait speed, even without nutritional supplementation. Shen et al. [11] similarly reported that multi-component training or its combination with nutrition was most effective for improving physical function in older adults, particularly when balance training was added to resistance programs. Their findings also showed that ART produced consistently larger improvements in function than RT alone, with moderate effect sizes.

### 4.3. Clinical Implications

This network meta-analysis is the first to systematically compare the relative efficacy of exercise intervention, protein-based nutritional supplementation, and their combination for key outcomes, including muscle mass, muscle strength, and physical function in middle-aged and older women with sarcopenia. The results showed that combined exercise and protein supplementation were more effective than either intervention alone across all primary outcomes. In particular, for physical function, the combined intervention may have clinically meaningful effects in improving usual gait speed. Protein supplementation alone did not show clear improvements in these outcomes among women with sarcopenia. Given that older women with sarcopenia often experience insufficient protein intake and marked functional limitations in clinical practice, we suggest that protein-based supplementation may be conditionally recommended as an adjunct strategy, provided it is implemented alongside a structured exercise program or reaches an adequate intake level (e.g., ≥1.11 g/kg/day), to help maintain daily function and delay functional dependence. For individuals who cannot tolerate high-dose protein intake, lower-burden alternatives such as amino acids or HMB may be considered.

To promote optimal neuromuscular adaptation, resistance training intensities of 70% to 85% of 1RM with a frequency of 2–3 sessions per week are generally recommended. However, the feasibility and cost-effectiveness of such programs may be limited for frail or functionally compromised older women, and the risk of adverse events may be elevated. Therefore, in clinical practice, especially for sarcopenic older women who are unable to tolerate high-intensity resistance training, it may be more appropriate to prioritize multi-component training strategies that are structurally diverse, functionally oriented, and practically feasible. Our findings highlight that multi-modal interventions such as ART or RBT, can exert significant benefits on physical function even in the absence of nutritional supplementation, underscoring their clinical value as feasible and effective alternatives.

### 4.4. Strengths and Limitations

In the context of previous meta-analyses primarily based on mixed-gender or male-dominant samples, this study provides the most comprehensive synthesis of current evidence regarding the effectiveness of exercise and protein supplementation in improving outcomes among middle-aged and older women with sarcopenia. Given that postmenopausal women often experience reduced protein metabolic efficiency, inadequate nutritional intake, and more severe functional impairments, sex-specific research is significant for developing precise intervention strategies. Methodologically, we adopted the minimally contextualized framework within the GRADE system. For specific key sarcopenia outcomes, we used MID as the threshold to interpret clinical relevance and grade the certainty of the evidence. In addition, we performed sensitivity analyses that excluded studies with a high risk of bias, studies with fewer than 15 participants per group, and studies that included individuals with possible sarcopenia. The results of these analyses support the robustness of our findings.

Despite these strengths, this study has some limitations. First, the quality of evidence was mainly very low to low. Some randomized controlled trials did not adequately report allocation concealment, which increased uncertainty in the risk of bias assessment. In addition, several trials had small sample sizes, which may have affected the stability of the effect estimates. Second, due to the limited number of included studies, this analysis did not account for total protein intake or specific protein types (such as whey, casein, or HMB), which may have contributed to heterogeneity in the results. Furthermore, although this study focused on protein-based supplementation, some included interventions also contained other components, which may have had synergistic effects on the outcomes. Future research should distinguish between different types and total amounts of protein intake and clarify their effects on muscle mass, strength, and physical function to provide more targeted and practical guidance for clinical practice.

## 5. Conclusions

This network meta-analysis showed that exercise intervention alone and with protein-based nutritional supplementation can effectively improve muscle mass, muscle strength, and physical function in middle-aged and older women with sarcopenia. In contrast, protein supplementation alone did not show significant benefits in these key outcomes, and some indicators of physical function even showed a downward trend. Based on these findings, a structured exercise intervention should be regarded as the core strategy, and protein supplementation may be added when conditions permit for the optimization of intervention effects and the delay of functional decline.

## Figures and Tables

**Table 1:** Basic characteristics of the included studies.

Study ID	Intervention	Age (Mean ± SD)	Sample Size	Duration (Weeks)	Detailed Intervention Description	Diagnostic Criteria	Country	Setting
Chen et al., 2018 [46]	Exercise	66.7 ± 5.3	17	8	Twice-weekly kettlebell training	AWGS	China	Community
	CG	68.3 ± 2.8	16	8	Maintained daily routine activities without structured intervention	AWGS	China	Community
Chen et al., 2023 [47]	Exercise	65.68 ± 2.5	25	8	Combined Tai Chi and resistance band training, performed three times per week	AWGS	China	Community
	CG	65.21 ± 2.6	24	8	Received health education only	AWGS	China	Community
Dieli-Conwright et al., 2018 [48]	Exercise	52.8 ± 10.6	50	16	Supervised combined aerobic and resistance exercise program, performed three times per week	AWGS	USA	Institution
	CG	53.6 ± 10.1	50	16	Maintained habitual lifestyle without additional intervention	AWGS	USA	Institution
El-Hak et al., 2021 [49]	Exercise	58.15 ± 3.06	20	12	Walking exercise performed three times per week	EWGSOP	Egypt	Institution
	Exercise	58.30 ± 2.81	20	12	Core muscle training and walking exercise, both performed three times per week	EWGSOP	Egypt	Institution
Huang et al., 2017 [50]	Exercise	68.89 ± 4.91	18	12	Progressive resistance training program using elastic bands	Study-defined criteria	China	Community
	CG	68.89 ± 4.91	17	12	Received health education only	Study-defined criteria	China	Community
Jung et al., 2024 [51]	Exercise	78.14 ± 3.72	14	12	Circuit training performed three times per week	AWGS	Korea	Community
	CG	78.21 ± 3.72	14	12	Maintained regular daily activities	AWGS	Korea	Community
Kim et al., 2012 [52]	Exercise + Nutrition	79.5 ± 2.9	38	12	Resistance plus balance training (twice weekly) combined with twice-daily supplementation of 3 g leucine-rich essential amino acids (6 g/day total)	Study-defined criteria	Japan	Community
	Nutrition	79.2 ± 2.8	39	12	Supplementation with 3 g leucine-rich essential amino acids, twice daily (6 g/day total)	Study-defined criteria	Japan	Community
	Exercise	79.0 ± 2.9	39	12	Resistance plus balance training performed twice weekly	Study-defined criteria	Japan	Community
	CG	78.7 ± 2.8	39	12	Received health education only	Study-defined criteria	Japan	Community
Kim et al., 2013 [53]	Exercise	79.6 ± 4.2	32	12	Same as above (resistance + balance training)	Study-defined criteria	Japan	Community
	CG	80.2 ± 5.6	32	12	Received health education only	Study-defined criteria	Japan	Community
Kim et al., 2016 [54]	Exercise + Nutrition	80.9 ± 4.2	36	12	Resistance and aerobic training (twice weekly) combined with essential amino acid supplementation (3 g/dose, twice daily, 6 g/day total)	Study-defined criteria	Japan	Community
	Exercise	81.4 ± 4.3	35	12	Resistance and aerobic training performed twice weekly	Study-defined criteria	Japan	Community
	Nutrition	81.2 ± 4.9	34	12	Supplementation with essential amino acids (3 g/dose, twice daily, 6 g/day total)	Study-defined criteria	Japan	Community
	CG	81.1 ± 5.1	34	12	Received health education only	Study-defined criteria	Japan	Community
Lee et al., 2021 [55]	Exercise	70.13 ± 4.51	15	12	Progressive elastic resistance band training performed three times per week	EWGSOP	China	Community
	CG	71.82 ± 5.33	12	12	Maintained usual daily activities	EWGSOP	China	Community
Liao et al., 2017 [56]	Exercise	68.42 ± 5.86	25	12	Elastic resistance band training	EWGSOP	China	Institution
	CG	66.39 ± 4.49	21	12	Maintained daily activity	EWGSOP	China	Institution
Liao et al., 2018 [57]	Exercise	66.67 ± 4.54	33	12	Elastic resistance band training	Study-defined criteria	China	Institution
	CG	68.32 ± 6.05	23	12	Maintained daily activity	Study-defined criteria	China	Institution
Liao et al., 2021 [58]	Exercise	69.81 ± 7.24	36	12	Elastic resistance band training twice weekly	AWGS	China	Institution
	Exercise + Nutrition	68.64 ± 7.42	36	12	Elastic resistance band training twice weekly combined with daily protein supplement (24.2 g/day: 11 g plant oligopeptides, 4 g casein peptides, 5 g BCAA), taken in two divided doses	AWGS	China	Institution
Nabuco et al., 2019 [59]	Exercise + Nutrition	68.0 ± 4.2	13	12	Daily supplementation with 35 g whey protein combined with resistance training three times per week	FNIH	Brazil	Community
	Exercise	70.1 ± 3.9	13	12	Daily placebo supplementation combined with the same resistance training protocol	FNIH	Brazil	Community
Osuka et al., 2021 [60]	Exercise + Nutrition	73.5 ± 4.2	36	12	Resistance training performed twice weekly combined with daily 1500 mg Ca-HMB supplementation	AWGS	Japan	Institution
	Exercise	71.8 ± 4.1	38	12	Resistance training performed twice per week	AWGS	Japan	Institution
	Nutrition	71.5 ± 4.5	37	12	Daily supplementation with 1500 mg Ca-HMB	AWGS	Japan	Institution
	CG	71.6 ± 4.2	38	12	Health education plus placebo supplementation	AWGS	Japan	Institution
Park et al., 2017 [61]	CG	74.7 ± 5.1	25	26	Health education only	Study-defined criteria	Korea	Community
	Exercise	73.5 ± 7.1	25	26	Combined aerobic and resistance training conducted five times per week	Study-defined criteria	Korea	Community
Rufino et al., 2023 [62]	Exercise	79.9 ± 7.2	20	26	High-intensity resistance training	EWGSOP	Spain	Community
	CG	79.6 ± 7.7	18	26	Maintained usual activity	EWGSOP	Spain	Community
Sammarco et al., 2017 [63]	CG	58 ± 10	9	16	Low-calorie placebo supplement	Study-defined criteria	Italy	Institution
	Nutrition	53 ± 8.9	9	16	Low-calorie, high-protein diet providing 1.2–1.4 g/kg/day of protein	Study-defined criteria	Italy	Institution
Seo et al., 2021 [64]	Exercise	70.3 ± 5.38	12	16	Bodyweight and resistance band exercises, three times per week	EWGSOP	Korea	Institution
	CG	72.9 ± 4.75	10	16	Maintained usual activity	EWGSOP	Korea	Institution
Valdés-Badilla et al., 2023 [65]	Exercise	73.91 ± 8.27	21	12	Progressive resistance band training, three times per week	EWGSOP	Chile	Community
	Exercise	72.85 ± 8.67	19	12	Structured group dance program, three times per week	EWGSOP	Chile	Community
Vasconcelos et al., 2016 [66]	Exercise	72 ± 4.6	14	10	Lower limb progressive resistance training, 2–3 times/week	Study-defined criteria	Brazil	Community
	CG	72 ± 3.6	14	10	Maintained daily activities	Study-defined criteria	Brazil	Community

**Table 2:** The results of the minimally contextualized framework.

Outcome	Certainty of Evidence	Group	Intervention	Intervention vs. Control	SUCRA
Handgrip strength	Low certainty (low to very low certainty evidence)	Category 2: among the most effective	Exercise + Nutrition	1.95 (0.10; 3.81)	74
			Exercise	1.85 (0.86; 2.85)	71.8
		Category 0: among the least effective	Nutrition	1.42 (−0.22; 3.05)	52.05
Knee extension strength	Low certainty (low to very low certainty evidence)	Category 2: among the most effective	Exercise	0.75 (0.41; 1.08)	84.58
			Exercise + Nutrition	0.71 (0.28; 1.14)	78.81
		Category 0: among the least effective	Nutrition	0.34 (−0.11; 0.79)	34.3
Usual gait speed	Low certainty (low to very low certainty evidence)	Category 2: among the most effective	Exercise + Nutrition	0.11 (0.04; 0.17)	94.49
			Exercise	0.08 (0.03; 0.12)	72.12
		Category 0: among the least effective	Nutrition	−0.04 (−0.11; 0.03)	5.11
Maximal gait speed	Low certainty (low to very low certainty evidence)	Category 2: among the most effective	Exercise + Nutrition	0.22 (0.06; 0.38)	82.17
			Exercise	0.21 (0.09; 0.33)	79.37
		Category 0: among the least effective	Nutrition	0.09 (−0.06; 0.25)	34.12
Appendicular skeletal muscle mass	High certainty (moderate to high certainty evidence)	Category 2: among the most effective	Exercise + Nutrition	0.21 (0.05; 0.38)	92.83
	Low certainty (low to very low certainty evidence)	Category 1: intermediately effective	Exercise	0.11 (−0.02; 0.23)	54.04
		Category 0: among the least effective	Nutrition	0.09 (−0.07; 0.26)	47.14
Skeletal muscle mass index	Low certainty (low to very low certainty evidence)	Category 0: among the least effective	Exercise + Nutrition	0.32 (−0.21; 0.85)	69.75
			Nutrition	0.29 (−0.35; 0.92)	61.71
			Exercise	0.22 (−0.11; 0.55)	55.2

Note: Intervention effectiveness was classified using a minimally contextualized framework, with the null value (MD = 0) as the threshold for comparison. Category 2 (among the most effective): The point estimate favors the intervention, and the 95% confidence interval (CI) excludes the null. Category 1 (intermediately effective): The point estimate favors the intervention, but the 95% CI includes or approaches the null. Category 0 (among the least effective): The point estimate is close to the null, and the 95% CI includes the null. Certainty of evidence was assessed using the GRADE framework and categorized as high/moderate or low/very low. SUCRA (surface under the cumulative ranking curve) reflects the probability of an intervention being among the most effective, ranging from 0 to 100%.

## Data Availability

All data generated or analyzed during this study are included in this published article (and its Supplementary Files).

## Supplementary Materials

The following supporting information can be downloaded at: https://www.mdpi.com/article/10.3390/nu17152392/s1, S1: Search strategy; S2: Risk of bias of randomized clinical trials; S3: Evaluation of inconsistency and heterogeneity; S4: Network forest plot; S5: League table; S6: CINeMA Assessment; S7: Funnel plots with Egger’s test for publication bias; S8: Subgroup analysis of different exercise modalities; S9: Sensitivity analysis.

## Abbreviations

The following abbreviations are used in this manuscript:

RCTs	Randomized controlled trials
MD	Mean difference
SD	Standard deviations
SMD	Standardized Mean Difference
CI	Confidence interval
MID	Minimal important difference
SMI	Skeletal muscle mass index
ASM	Appendicular skeletal muscle mass
RT	Resistance training
RT + Nu	Resistance training and nutrition
RBT	Resistance balance training
ART	Aerobic resistance training
