# Supplementary material for: Comparative Effectiveness of Exercise, Protein Supplementation, and Combined Interventions for Sarcopenia Management in Women: A Network Meta-Analysis
Source: Nutrients. 2025 Jul 22;17(15):2392. doi: 10.3390/nu17152392 (PMC12348085; doi:10.3390/nu17152392)

Supplementary Material

Comparative Effectiveness of Exercise, Protein Supplementation, and Combined Interventions for Sarcopenia Management in Women: A Network Meta-Analysis

Table of contents

Section S1: Search strategy 2

Section S2: Risk of bias of randomized clinical trials 9

Section S3: Evaluation of inconsistency and heterogeneity 10

Section S4: Network forest plot 13

Section S5: League table 19

Section S6: CINeMA Assessment 22

Section S7: Funnel Plots with Egger's Test for Publication Bias 29

Section S8: Subgroup Analysis of Different Exercise Modalities 32

Section S9: Sensitivity analysis 44

Section S1: Search strategy

Table S1 Search strategy of Pubmed

#	Searches
1	(((((((((((((((((((((((((Exercises[Title/Abstract]) OR (Exercise,Physical[Title/Abstract])) OR (Exercises, Physical[Title/Abstract])) OR (Physical Exercise[Title/Abstract])) OR (Physical Exercises[Title/Abstract])) OR (Physical Activity[Title/Abstract])) OR (Activities, Physical[Title/Abstract])) OR (Activity, Physical[Title/Abstract])) OR (Physical Activities[Title/Abstract])) OR (Exercise, Aerobic[Title/Abstract])) OR (Aerobic Exercise[Title/Abstract])) OR (Aerobic Exercises[Title/Abstract])) OR (Exercises, Aerobic[Title/Abstract])) OR (Exercise, Isometric[Title/Abstract])) OR (Exercises, Isometric[Title/Abstract])) OR (Isometric Exercises[Title/Abstract])) OR (Isometric Exercise[Title/Abstract])) OR (Acute Exercise[Title/Abstract])) OR (Acute Exercises[Title/Abstract])) OR (Exercise, Acute[Title/Abstract])) OR (Exercises, Acute[Title/Abstract])) OR (Exercise Training[Title/Abstract])) OR (Exercise Trainings[Title/Abstract])) OR (Training, Exercise[Title/Abstract])) OR (Trainings, Exercise[Title/Abstract])) OR (Exercise[MeSH Terms])
2	(((((((((((((((((((((((Training, Resistance[Title/Abstract]) OR (Strength Training[Title/Abstract])) OR (Training, Strength[Title/Abstract])) OR (Weight-Lifting Strengthening Program[Title/Abstract])) OR (Strengthening Programs, Weight-Lifting[Title/Abstract])) OR (Strengthening Program, Weight-Lifting[Title/Abstract])) OR (Weight Lifting Strengthening Program[Title/Abstract])) OR (Weight-Lifting Strengthening Programs[Title/Abstract])) OR (Weight-Lifting Exercise Program[Title/Abstract])) OR (Exercise Programs, Weight-Lifting[Title/Abstract])) OR (Exercise Program, Weight-Lifting[Title/Abstract])) OR (Weight Lifting Exercise Program[Title/Abstract])) OR (Weight-Lifting Exercise Programs[Title/Abstract])) OR (Weight-Bearing Strengthening Program[Title/Abstract])) OR (Strengthening Programs, Weight-Bearing[Title/Abstract])) OR (Strengthening Program, Weight-Bearing[Title/Abstract])) OR (Weight Bearing Strengthening Program[Title/Abstract])) OR (Weight-Bearing Strengthening Programs[Title/Abstract])) OR (Weight-Bearing Exercise Program[Title/Abstract])) OR (Exercise Programs, Weight-Bearing[Title/Abstract])) OR (Exercise Program, Weight-Bearing[Title/Abstract])) OR (Weight Bearing Exercise Program[Title/Abstract])) OR (Weight-Bearing Exercise Programs[Title/Abstract])) OR (Resistance training[MeSH Terms])
3	((((((((Cardiovascular Exercise[Title/Abstract]) OR (Endurance Training[Title/Abstract])) OR (Walking[Title/Abstract])) OR (Running[Title/Abstract])) OR (Cycling[Title/Abstract])) OR (Swimming[Title/Abstract])) OR (Dancing[Title/Abstract])) OR (combined training[Title/Abstract])) OR (Concurrent Training[Title/Abstract])
4	((((((((((((High Intensity Interval Training[Title/Abstract]) OR (High-Intensity Interval Trainings[Title/Abstract])) OR (Interval Training, High-Intensity[Title/Abstract])) OR (Interval Trainings, High-Intensity[Title/Abstract])) OR (Training, High-Intensity Interval[Title/Abstract])) OR (Trainings, High-Intensity Interval[Title/Abstract])) OR (High-Intensity Intermittent Exercise[Title/Abstract])) OR (Exercise, High-Intensity Intermittent[Title/Abstract])) OR (Exercises, High-Intensity Intermittent[Title/Abstract])) OR (High-Intensity Intermittent Exercises[Title/Abstract])) OR (Sprint Interval Training[Title/Abstract])) OR (Sprint Interval Trainings[Title/Abstract])) OR (High-Intensity Interval Training[MeSH Terms])
5	((((((Circuit-Based Exercise[MeSH Terms]) OR (Circuit Based Exercise[Title/Abstract])) OR (Circuit-Based Exercises[Title/Abstract])) OR (Exercise, Circuit-Based[Title/Abstract])) OR (Exercises, Circuit-Based[Title/Abstract])) OR (Circuit Training[Title/Abstract])) OR (Training, Circuit[Title/Abstract])
6	((((((((((((((((Chinese exercise[Title/Abstract]) OR (traditional exercise[Title/Abstract])) OR (traditional chinese medicine[Title/Abstract])) OR (chinese traditional exercise[Title/Abstract])) OR (traditional chinese exercise[Title/Abstract])) OR (traditional exercise[Title/Abstract])) OR (traditional Kungfu[Title/Abstract])) OR (Kungfu[Title/Abstract])) OR (Shadowboxing[Title/Abstract])) OR (Five-animal exercises[Title/Abstract])) OR (Wuqinxi[Title/Abstract])) OR (Five animal frolics[Title/Abstract])) OR (Five-animal boxing[Title/Abstract])) OR (Baduanjin[Title/Abstract])) OR (Yijinjing[Title/Abstract])) OR (liuzijue[Title/Abstract])) OR (((Qigong[MeSH Terms]) OR (Ch'i Kung[Title/Abstract])) OR (Qi Gong[Title/Abstract]))) OR (((((((((taiji[MeSH Terms]) OR (Tai-ji[Title/Abstract])) OR (Tai Chi[Title/Abstract])) OR (Chi, Tai[Title/Abstract])) OR (Tai Chi Chuan[Title/Abstract])) OR (Taijiquan[Title/Abstract])) OR (T'ai Chi[Title/Abstract])) OR (Tai Ji Quan[Title/Abstract])) OR (Ji Quan, Tai[Title/Abstract])) OR (Quan, Tai Ji[Title/Abstract]))

7	(((Mind-body exercises[Title/Abstract]) OR (Yoga[MeSH Terms])) OR (dance[Title/Abstract])) OR (Pilates[Title/Abstract])
8	((((((Balance training[Title/Abstract]) OR (Balance exercise[Title/Abstract])) OR (Balance intervention[Title/Abstract])) OR (Postural control[Title/Abstract])) OR (Stability training[Title/Abstract])) OR (Proprioception training[Title/Abstract])) OR (Motor control training[Title/Abstract])
9	(((((Blood Flow Restriction Therapy[MeSH Terms]) OR (Blood Flow Restriction Training[Title/Abstract])) OR (Blood Flow Restriction Exercise[Title/Abstract])) OR (BFR Therapy[Title/Abstract])) OR (BFR Therapies[Title/Abstract])) OR (Therapy, BFR[Title/Abstract])
10	(((Sarcopenia[MeSH Terms]) OR (sarcopenia[Title/Abstract])) OR (Muscle loss[Title/Abstract])) OR (Sarcopenias[Title/Abstract])
11	(((((((((((Whey Proteins[MeSH Terms]) OR (Proteins, Whey[Title/Abstract])) OR (Protein, Whey[Title/Abstract])) OR (Whey Protein[Title/Abstract])) OR (whey supplementation[Title/Abstract])) OR (whey protein supplementation[Title/Abstract])) OR (whey intake[Title/Abstract])) OR (whey protein isolate[Title/Abstract])) OR (whey protein concentrate[Title/Abstract])) OR (milk proteins[Title/Abstract])) OR (milk protein[Title/Abstract])) OR (dairy proteins[Title/Abstract])) OR (dairy protein[Title/Abstract])
12	(((((((Protein[MeSH Terms]) OR (Dietary Supplements[MeSH Terms])) OR (Amino Acids[MeSH Terms])) OR (Dietary Supplement[Title/Abstract])) OR (Protein supplement[Title/Abstract])) OR (Supplements, Dietary[Title/Abstract])) OR (Dietary Supplementations[Title/Abstract])) OR (Supplementations, Dietary[Title/Abstract])
13	((((((beta-hydroxyisovaleric acid[MeSH Terms]) OR (beta hydroxy beta methylbutyrate[Title/Abstract])) OR (beta-hydroxy beta-methylbutyrate[Title/Abstract])) OR (3-hydroxyisovaleric acid[Title/Abstract])) OR (beta-hydroxy-beta-methylbutyrate[Title/Abstract])) OR (HMB-d6[Title/Abstract])) OR (HMB[Title/Abstract])
14	((((((Amino Acids, Essential[MeSH Terms]) OR (Acids, Essential Amino[Title/Abstract])) OR (Essential Amino Acid[Title/Abstract])) OR (Acid, Essential Amino[Title/Abstract])) OR (Amino Acid, Essential[Title/Abstract])) OR (Essential Amino Acids[Title/Abstract])) OR (EAA[Title/Abstract])
15	((((Leucine[MeSH Terms]) OR (Leucine, L-Isomer[Title/Abstract])) OR (Leucine, L Isomer[Title/Abstract])) OR (L-Isomer Leucine[Title/Abstract])) OR (L-Leucine[Title/Abstract])
16	((((((((Amino Acids, Branched-Chain[MeSH Terms]) OR (Acids, Branched-Chain Amino[Title/Abstract])) OR (Branched-Chain Amino Acids[Title/Abstract])) OR (Branched-Chain Amino Acid[Title/Abstract])) OR (Acid, Branched-Chain Amino[Title/Abstract])) OR (Amino Acid, Branched-Chain[Title/Abstract])) OR (Branched Chain Amino Acid[Title/Abstract])) OR (Amino Acids, Branched Chain[Title/Abstract])) OR (BCAA[Title/Abstract])
17	((((((((((((Therapy, Nutrition[MeSH Terms]) OR (Therapy, Nutrition[Title/Abstract])) OR (Medical Nutrition Therapy[Title/Abstract])) OR (Nutrition Therapy, Medical[Title/Abstract])) OR (Therapy, Medical Nutrition[Title/Abstract])) OR (Diet Therapy[MeSH Terms])) OR (Diet Therapies[Title/Abstract])) OR (Therapy, Diet[Title/Abstract])) OR (Dietary Modification[Title/Abstract])) OR (Dietary Modifications[Title/Abstract])) OR (Diet Modification[Title/Abstract])) OR (Diet Modifications[Title/Abstract])) OR (Modification, Diet[Title/Abstract])) OR (Diet, Food, and Nutrition[MeSH Terms])
18	(Women[MeSH Terms]) OR (Female[MeSH Terms])
19	(((((randomized controlled trial[Publication Type]) OR (randomized)) OR (clinical trials as topic[MeSH Terms])) OR (placebo)) OR (randomly)) OR (trial) OR (randomised)
20	#1 OR #2 OR #3 OR #4 OR #5 OR #6 OR #7 OR #8 OR #9 OR #11 OR #12 OR #13 OR #14 OR #15 OR #16 OR #17
19	#10 AND #18 AND #19 AND #20

Table S2 Search strategy of Web of Science

#	Searches
1	TS=("Sarcopenia" OR "Sarcopenias" OR "Muscle loss")
2	TS=("Exercise" OR "Exercises" OR "Exercise, Physical" OR "Exercises, Physical" OR "Physical Exercise" OR "Physical Exercises" OR "Physical Activity" OR "Activities, Physical" OR "Activity, Physical" OR "Physical Activities" OR "Exercise, Aerobic" OR "Aerobic Exercise" OR "Aerobic Exercises" OR "Exercises, Aerobic" OR "Exercise, Isometric" OR "Exercises, Isometric" OR "Isometric Exercises" OR "Isometric Exercise" OR "Acute Exercise" OR "Acute Exercises" OR "Exercise, Acute" OR "Exercises, Acute" OR "Exercise Training" OR "Exercise Trainings" OR "Training, Exercise" OR "Trainings, Exercise")
3	TS=("Resistance training" OR "Training, Resistance" OR "Strength Training" OR "Training, Strength" OR "Weight-Lifting Strengthening Program" OR "Strengthening Programs, Weight-Lifting" OR "Strengthening Program, Weight-Lifting" OR "Weight Lifting Strengthening Program" OR "Weight-Lifting Strengthening Programs" OR "Weight-Lifting Exercise Program" OR "Exercise Programs, Weight-Lifting" OR "Exercise Program, Weight-Lifting" OR "Weight Lifting Exercise Program" OR "Weight-Lifting Exercise Programs" OR "Weight-Bearing Strengthening Program" OR "Strengthening Programs, Weight-Bearing" OR "Strengthening Program, Weight-Bearing" OR "Weight Bearing Strengthening Program" OR "Weight-Bearing Strengthening Programs" OR "Weight-Bearing Exercise Program" OR "Exercise Programs, Weight-Bearing" OR "Exercise Program, Weight-Bearing" OR "Weight Bearing Exercise Program" OR "Weight-Bearing Exercise Programs")
4	TS=("Cardiovascular Exercise" OR "Endurance Training" OR "Walking" OR "Running" OR "Cycling" OR "Swimming" OR "Dancing" OR "combined training" OR "Concurrent Training")
5	TS=("High-Intensity Interval Training" OR "High Intensity Interval Training" OR "High-Intensity Interval Trainings" OR "Interval Training, High-Intensity" OR "Interval Trainings, High-Intensity" OR "Training, High-Intensity Interval" OR "Trainings, High-Intensity Interval" OR "High-Intensity Intermittent Exercise" OR "Exercise, High-Intensity Intermittent" OR "Exercises, High-Intensity Intermittent" OR "High-Intensity Intermittent Exercises" OR "Sprint Interval Training" OR "Sprint Interval Trainings")
6	TS=("Circuit-Based Exercise" OR "Circuit Based Exercise" OR "Circuit-Based Exercises" OR "Exercise, Circuit-Based" OR "Exercises, Circuit-Based" OR "Circuit Training" OR "Training, Circuit")
7	TS=("Chinese exercise" OR "traditional exercise" OR "traditional chinese medicine" OR "chinese traditional exercise" OR "traditional chinese exercise" OR "traditional Kungfu" OR "Kungfu" OR "Shadowboxing" OR "Five-animal exercises" OR "Wuqinxi" OR "Five animal frolics" OR "Five-animal boxing" OR "Baduanjin" OR "Yijinjing" OR "liuzijue" OR "Qigong" OR "Ch'i Kung" OR "Qi Gong" OR "taiji" OR "Tai-ji" OR "Tai Chi" OR "Chi, Tai" OR "Tai Chi Chuan" OR "Taijiquan" OR "T'ai Chi" OR "Tai Ji Quan" OR "Ji Quan, Tai" OR "Quan, Tai Ji")
8	TS=("Mind-body exercises" OR "yoga" OR "dance" OR "Pilates")
9	TS=("Balance training" OR "Balance exercise" OR "Balance intervention" OR "Postural control" OR "Stability training" OR "Proprioception training" OR "Motor control training")
10	TS=("Blood Flow Restriction Therapy" OR "Blood Flow Restriction Training" OR "Blood Flow Restriction Exercise" OR "BFR Therapy" OR "BFR Therapies" OR "Therapy, BFR")
11	TS=("Protein" OR "Dietary Supplements" OR "Amino Acids" OR "Dietary Supplement" OR "Protein supplement" OR "Supplements, Dietary" OR "Dietary Supplementations" OR "Supplementations, Dietary")
12	TS=("Whey Proteins" OR "Proteins, Whey" OR "Protein, Whey" OR "Whey Protein" OR "whey supplementation" OR "whey protein supplementation" OR "whey intake" OR "whey protein isolate" OR "whey protein concentrate" OR "milk proteins" OR "milk protein" OR "dairy proteins" OR "dairy protein")
13	TS=("Amino Acids, Essential" OR "Acids, Essential Amino" OR "Essential Amino Acid" OR "Acid, Essential Amino" OR "Amino Acid, Essential" OR "Essential Amino Acids" OR "EAA")
14	TS=("Leucine" OR "Leucine, L-Isomer" OR "Leucine, L Isomer" OR "L-Isomer Leucine" OR "L-Leucine")
15	TS=("Therapy, Nutrition" OR "Medical Nutrition Therapy" OR "Nutrition Therapy, Medical" OR "Therapy, Medical Nutrition" OR "Diet Therapy" OR "Diet Therapies" OR "Therapy, Diet" OR "Dietary Modification" OR "Dietary Modifications" OR "Diet Modification" OR "Diet Modifications" OR "Modification, Diet" OR "Diet, Food, and Nutrition")

16	TS=("beta-hydroxyisovaleric acid" OR "beta hydroxy beta methylbutyrate" OR "beta-hydroxy beta-methylbutyrate" OR "3-hydroxyisovaleric acid" OR "beta-hydroxy-beta-methylbutyrate" OR "HMB-d6" OR "HMB")
17	TS=("Amino Acids, Branched-Chain" OR "Acids, Branched-Chain Amino" OR "Branched-Chain Amino Acids" OR "Branched-Chain Amino Acid" OR "Acid, Branched-Chain Amino" OR "Amino Acid, Branched-Chain" OR "Branched Chain Amino Acid" OR "Amino Acids, Branched Chain" OR "BCAA")
18	TS=("Women" OR "Female")
19	TS=("randomized controlled trial" OR "randomized" OR "clinical trials" OR "placebo" OR "randomly" OR "trial" OR "randomised")
20	#2 OR #3 OR #4 OR #5 OR #6 OR #7 OR #8 OR #9 OR #10 OR #11 OR #12 OR #13 OR #14 OR #15 OR #16 OR #17
21	#1 AND #18 AND #19 AND #20

Table S3 Search strategy of Cochrane Central Register of Controlled Trials

#	Searches
1	MeSH descriptor: [Sarcopenia] explode all trees
2	MeSH descriptor: [High-Intensity Interval Training] explode all trees
3	MeSH descriptor: [Exercise] explode all trees
4	MeSH descriptor: [Circuit-Based Exercise] explode all trees
5	MeSH descriptor: [Resistance Training] explode all trees
6	MeSH descriptor: [Tai Ji] explode all trees
7	MeSH descriptor: [Qigong] explode all trees
8	MeSH descriptor: [Yoga] explode all trees
9	MeSH descriptor: [Exercise Movement Techniques] explode all trees
10	MeSH descriptor: [Blood Flow Restriction Therapy] explode all trees
11	("Balance training":ti,ab,kw OR "Balance exercise":ti,ab,kw OR "Balance intervention":ti,ab,kw OR "Postural control":ti,ab,kw OR "Stability training":ti,ab,kw OR "Proprioception training":ti,ab,kw OR "Motor control training":ti,ab,kw)
12	("aerobic exercise":ti,ab,kw OR "aerobic training":ti,ab,kw OR "Cardiovascular Exercise":ti,ab,kw OR "Endurance Training":ti,ab,kw OR "Walking":ti,ab,kw OR "Running":ti,ab,kw OR "Cycling":ti,ab,kw OR "Swimming":ti,ab,kw OR "Dancing":ti,ab,kw OR "combined training":ti,ab,kw OR "Concurrent Training":ti,ab,kw OR "Mind-body exercises":ti,ab,kw OR "yoga":ti,ab,kw OR "dance":ti,ab,kw OR "pilates":ti,ab,kw OR "traditional chinese medicine":ti,ab,kw OR "chinese traditional exercise":ti,ab,kw OR "traditional exercise":ti,ab,kw OR "traditional Kungfu":ti,ab,kw OR "Kungfu":ti,ab,kw OR "Shadowboxing":ti,ab,kw OR "Wuqinxi":ti,ab,kw OR "Five-animal exercises":ti,ab,kw OR "Five animal frolics":ti,ab,kw OR "Five-animal boxing":ti,ab,kw OR "Baduanjin":ti,ab,kw OR "Yijinjing":ti,ab,kw OR "liuzijue":ti,ab,kw)
13	MeSH descriptor: [Proteins] explode all trees
14	MeSH descriptor: [Amino Acids] explode all trees
15	MeSH descriptor: [Whey Proteins] explode all trees
16	MeSH descriptor: [beta-hydroxyisovaleric acid] explode all trees
17	MeSH descriptor: [Amino Acids, Essential] explode all trees
18	MeSH descriptor: [Leucine] explode all trees
19	MeSH descriptor: [Therapy, Nutrition] explode all trees
20	MeSH descriptor: [Diet Therapy] explode all trees
21	MeSH descriptor: [Diet, Food, and Nutrition] explode all trees
22	MeSH descriptor: [Amino Acids, Branched-Chain] explode all trees
23	MeSH descriptor: [Female] explode all trees
24	MeSH descriptor: [Women] explode all trees
25	#2 OR #3 OR #4 OR #5 OR #6 OR #7 OR #8 OR #9 OR #10 OR #11 OR #12 OR #13 OR #14 OR #15 OR #16 OR #17 OR #18 OR #19 OR #20 OR #21 OR #22
26	#23 OR #24
27	#1 AND #25 AND #26

Table S4 Search strategy of Embase

#	Searches
1	'Sarcopenia'/exp OR sarcopenia:ab,ti OR 'Muscle loss':ab,ti OR sarcopenias:ab,ti
2	'Exercise'/exp OR 'Exercise':ab,ti OR 'Exercises':ab,ti OR 'Exercise, Physical':ab,ti OR 'Exercises, Physical':ab,ti OR 'Physical Exercise':ab,ti OR 'Physical Exercises':ab,ti OR 'Physical Activity':ab,ti OR 'Activities, Physical':ab,ti OR 'Activity, Physical':ab,ti OR 'Physical Activities':ab,ti OR 'Exercise, Aerobic':ab,ti OR 'Aerobic Exercise':ab,ti OR 'Aerobic Exercises':ab,ti OR 'Exercises, Aerobic':ab,ti OR 'Exercise, Isometric':ab,ti OR 'Exercises, Isometric':ab,ti OR 'Isometric Exercises':ab,ti OR 'Isometric Exercise':ab,ti OR 'Acute Exercise':ab,ti OR 'Acute Exercises':ab,ti OR 'Exercise, Acute':ab,ti OR 'Exercises, Acute':ab,ti OR 'Exercise Training':ab,ti OR 'Exercise Trainings':ab,ti OR 'Training, Exercise':ab,ti OR 'Trainings, Exercise':ab,ti
3	'Resistance training'/exp OR 'Resistance training':ab,ti OR 'Training, Resistance':ab,ti OR 'Strength Training':ab,ti OR 'Training, Strength':ab,ti OR 'Weight-Lifting Strengthening Program':ab,ti OR 'Strengthening Programs, Weight-Lifting':ab,ti OR 'Strengthening Program, Weight-Lifting':ab,ti OR 'Weight Lifting Strengthening Program':ab,ti OR 'Weight-Lifting Strengthening Programs':ab,ti OR 'Weight-Lifting Exercise Program':ab,ti OR 'Exercise Programs, Weight-Lifting':ab,ti OR 'Exercise Program, Weight-Lifting':ab,ti OR 'Weight Lifting Exercise Program':ab,ti OR 'Weight-Lifting Exercise Programs':ab,ti OR 'Weight-Bearing Strengthening Program':ab,ti OR 'Strengthening Programs, Weight-Bearing':ab,ti OR 'Strengthening Program, Weight-Bearing':ab,ti OR 'Weight Bearing Strengthening Program':ab,ti OR 'Weight-Bearing Strengthening Programs':ab,ti OR 'Weight-Bearing Exercise Program':ab,ti OR 'Exercise Programs, Weight-Bearing':ab,ti OR 'Exercise Program, Weight-Bearing':ab,ti OR 'Weight Bearing Exercise Program':ab,ti OR 'Weight-Bearing Exercise Programs':ab,ti
4	'Cardiovascular Exercise':ab,ti OR 'Endurance Training':ab,ti OR 'Walking':ab,ti OR 'Running':ab,ti OR 'Cycling':ab,ti OR 'Swimming':ab,ti OR 'Dancing':ab,ti OR 'combined training':ab,ti OR 'Concurrent Training':ab,ti
5	'High-Intensity Interval Training'/exp OR 'High Intensity Interval Training':ab,ti OR 'High-Intensity Interval Trainings':ab,ti OR 'Interval Training, High-Intensity':ab,ti OR 'Interval Trainings, High-Intensity':ab,ti OR 'Training, High-Intensity Interval':ab,ti OR 'Trainings, High-Intensity Interval':ab,ti OR 'High-Intensity Intermittent Exercise':ab,ti OR 'Exercise, High-Intensity Intermittent':ab,ti OR 'Exercises, High-Intensity Intermittent':ab,ti OR 'High-Intensity Intermittent Exercises':ab,ti OR 'Sprint Interval Training':ab,ti OR 'Sprint Interval Trainings':ab,ti
6	'Circuit-Based Exercise'/exp OR 'Circuit Based Exercise':ab,ti OR 'Circuit-Based Exercises':ab,ti OR 'Exercise, Circuit-Based':ab,ti OR 'Exercises, Circuit-Based':ab,ti OR 'Circuit Training'/exp OR 'Training, Circuit':ab,ti
7	'chinese exercise':ab,ti OR 'traditional exercise':ab,ti OR 'traditional chinese medicine':ab,ti OR 'chinese traditional exercise':ab,ti OR 'traditional chinese exercise':ab,ti OR 'traditional kungfu':ab,ti OR 'kungfu':ab,ti OR 'shadowboxing':ab,ti OR 'five-animal exercises':ab,ti OR 'wuqinxi':ab,ti OR 'five animal frolics':ab,ti OR 'five-animal boxing':ab,ti OR 'baduanjin':ab,ti OR 'yijinjing':ab,ti OR 'liuzijue':ab,ti OR 'qigong'/exp OR 'chi kung':ab,ti OR 'qi gong':ab,ti OR 'taiji' OR 'tai-ji':ab,ti OR 'chi, tai':ab,ti OR 'tai chi chuan':ab,ti OR 'taijiquan':ab,ti OR 'tai chi':ab,ti OR 'tai ji quan':ab,ti OR 'ji quan, tai':ab,ti OR 'quan, tai ji':ab,ti
8	'Mind-body exercises':ab,ti OR 'yoga'/exp OR 'dance':ab,ti OR 'Pilates'/exp
9	('Balance training':ab,ti OR 'Balance exercise':ab,ti OR 'Balance intervention':ab,ti OR 'Postural control':ab,ti OR 'Stability training':ab,ti OR 'Proprioception training':ab,ti OR 'Motor control training':ab,ti)
10	('Blood Flow Restriction Therapy'/exp OR 'Blood Flow Restriction Training':ab,ti OR 'Blood Flow Restriction Exercise':ab,ti OR 'BFR Therapy':ab,ti OR 'BFR Therapies':ab,ti OR 'Therapy, BFR':ab,ti)
11	'Whey Proteins'/exp OR 'Proteins, Whey':ab,ti OR 'Protein, Whey':ab,ti OR 'Whey Protein':ab,ti OR 'whey supplementation':ab,ti OR 'whey protein supplementation':ab,ti OR 'whey intake':ab,ti OR 'whey protein isolate':ab,ti OR 'whey protein concentrate':ab,ti OR 'milk proteins':ab,ti OR 'milk protein':ab,ti OR 'dairy proteins':ab,ti OR 'dairy protein':ab,ti
12	'beta-hydroxyisovaleric acid'/exp OR 'beta hydroxy beta methylbutyrate':ab,ti OR 'beta-hydroxy beta-methylbutyrate':ab,ti OR '3-hydroxyisovaleric acid':ab,ti OR 'beta-hydroxy-beta-methylbutyrate':ab,ti OR 'HMB-d6':ab,ti OR 'HMB':ab,ti
13	'Amino Acids, Essential'/exp OR 'Acids, Essential Amino':ab,ti OR 'Essential Amino Acid':ab,ti OR 'Acid, Essential Amino':ab,ti OR 'Amino Acid, Essential':ab,ti OR 'Essential Amino Acids':ab,ti OR 'EAA':ab,ti
14	'Leucine'/exp OR 'Leucine, L-Isomer':ab,ti OR 'Leucine, L Isomer':ab,ti OR 'L-Isomer Leucine':ab,ti OR 'L-Leucine':ab,ti

15	'Amino Acids, Branched-Chain'/exp OR 'Acids, Branched-Chain Amino':ab,ti OR 'Branched-Chain Amino Acids':ab,ti OR 'Branched-Chain Amino Acid':ab,ti OR 'Acid, Branched-Chain Amino':ab,ti OR 'Amino Acid, Branched-Chain':ab,ti OR 'Branched Chain Amino Acid':ab,ti OR 'Amino Acids, Branched Chain':ab,ti OR 'BCAA':ab,ti
16	'Therapy, Nutrition'/exp OR 'Therapy, Nutrition':ab,ti OR 'Medical Nutrition Therapy':ab,ti OR 'Nutrition Therapy, Medical':ab,ti OR 'Therapy, Medical Nutrition':ab,ti OR 'Diet Therapy'/exp OR 'Diet Therapies':ab,ti OR 'Therapy, Diet':ab,ti OR 'Dietary Modification':ab,ti OR 'Dietary Modifications':ab,ti OR 'Diet Modification':ab,ti OR 'Diet Modifications':ab,ti OR 'Modification, Diet':ab,ti OR 'Diet, Food, and Nutrition'/exp
17	'dietary supplement'/exp OR 'dietary supplement':ab,ti OR 'protein supplement':ab,ti OR 'supplements, dietary':ab,ti OR 'dietary supplementations':ab,ti OR 'supplementations, dietary':ab,ti OR 'protein'/exp OR 'protein':ab,ti OR 'amino acid'/exp OR 'amino acid':ab,ti
18	'randomized controlled trial'/exp OR 'randomized':ab,ti OR 'clinical trials':ab,ti OR 'placebo':ab,ti OR 'randomly':ab,ti OR 'trial':ab,ti OR 'randomised':ab,ti
19	'Female'/exp OR 'Women'/exp
20	#2 OR #3 OR #4 OR #5 OR #6 OR #7 OR #8 OR #9 OR #10 #11 OR #12 OR #13 OR #14 OR #15 OR #16 OR #17
21	#1 AND #18 AND #19 AND #20

Section S2: Risk of bias of randomized clinical trials

Table S5: Study level risk of bias assessment using Cochrane risk of bias tool 2.0 for assessing risk of bias of randomized clinical trials.

Unique ID	Randomization process	Deviations from intended interventions	Missing outcome data	Measurement of the outcome	Selection of the reported result	Overall Bias
Chen et al 2018	Some concerns	Low	Low	Low	Some concerns	Some concerns
Chen et al2023	Low	Low	Low	Low	Some concerns	Some concerns
Dieli-Conwright et al2018	Low	Low	Low	Low	Low	Low
EI-Hak et al2021	Some concerns	Some concerns	Low	Low	Some concerns	Some concerns
Huang et al2017	Low	Low	Low	Low	Low	Low
Jung et al2024	Some concerns	Some concerns	Low	Low	Some concerns	Some concerns
Kim et al2012	Some concerns	Low	Low	Low	Some concerns	Some concerns
Kim et al2013	Low	Low	Low	Low	Low	Low
Kim et al2016	Some concerns	Some concerns	Low	Low	Some concerns	Some concerns
Lee et al2021	Low	Low	Low	Low	Low	Low
Liao et al 2017	Some concerns	Low	Low	Low	Some concerns	Some concerns
Liao et al2018	Low	Low	Low	Low	Low	Low
Liao et al2021	Low	Low	Low	Low	Low	Low
Nabuco et al2019	Some concerns	Low	Low	Low	Low	Some concerns
Osuka et al2021	Low	Low	Low	Low	Low	Low
Park et al2017	Some concerns	Low	Low	Low	Some concerns	Some concerns
Rufino et al2023	Low	Low	Low	Low	Low	Low
Sammarco et al2017	High	Some concerns	Low	Low	Some concerns	High
Seo et al2021	Some concerns	Low	Low	Low	Some concerns	Some concerns
Valdés-Badilla et al2023	Low	Low	Low	Low	Low	Low
Vasconcelos et al2016	Low	Low	Low	Low	Low	Low

Section S3: Evaluation of inconsistency and heterogeneity

Table S6: Global consistency and heterogeneity

**Clinical outcome**	**Chi square**	**P value**	I^2	τ^2
Handgrip strength	3.71	0.1563	63.7%	1.3422
Knee extension strength	1.83	0.3998	63.9%	0.1252
Usual gait speed	1.61	0.4473	51.3%	0.0028
Maximal gait speed	0.08	0.7815	71.5%	0.0113
ASM	1.42	0.4913	0	0
SMI	1.97	0.3731	49.2%	0.00804

Table S7: Side-splitting of Handgrip strength. Inconsistency test between direct and indirect treatment comparisons in mixed treatment comparison.

comparison	k	prop	nma	direct	indir.	Diff	z	p-value
Exercise:CG	10	0.99	1.8532	1.8487	2.1806	-0.3319	-0.08	0.94
Exercise+Nutrition:CG	2	0.81	1.952	1.1	5.6198	-4.5198	-1.87	0.0617
Nutrition:CG	3	0.84	1.416	0.7539	5.0054	-4.2516	-1.85	0.0647
Exercise:Exercise+Nutrition	2	0.84	-0.0988	-0.8863	3.9071	-4.7935	-1.87	0.0614
Exercise:Nutrition	2	0.73	0.4372	-0.3361	2.5248	-2.8609	-1.49	0.1372
Exercise+Nutrition:Nutrition	2	0.95	0.536	0.5443	0.3773	0.167	0.04	0.972

Table S8: Side-splitting of Knee extension strength. Inconsistency test between direct and indirect treatment comparisons in mixed treatment comparison.

comparison	k	prop	nma	direct	indir.	Diff	z	p-value
Exercise:CG	7	0.98	0.7487	0.7542	0.5064	0.2478	0.21	0.8308
Exercise+Nutrition:CG	3	0.8	0.7099	0.5684	1.2828	-0.7144	-1.29	0.1973
Nutrition:CG	3	0.87	0.3403	0.2099	1.224	-1.0141	-1.48	0.1386
Exercise:Exercise+Nutrition	4	0.9	0.0388	-0.0685	0.9589	-1.0273	-1.49	0.1363
Exercise:Nutrition	3	0.85	0.4084	0.2885	1.0804	-0.7919	-1.25	0.2098
Exercise+Nutrition:Nutrition	3	0.95	0.3696	0.3595	0.5793	-0.2198	-0.19	0.8483

Table S9: Side-splitting of Usual gait speed. Inconsistency test between direct and indirect treatment comparisons in mixed treatment comparison.

comparison	k	prop	nma	direct	indir.	Diff	z	p-value
Exercise:CG	12	0.97	0.0776	0.0794	0.0273	0.0521	0.41	0.6825
Exercise+Nutrition:CG	3	0.64	0.1074	0.1202	0.0844	0.0358	0.52	0.6063
Nutrition:CG	3	0.82	-0.0371	-0.0178	-0.1232	0.1054	1.13	0.2602
Exercise:Exercise+Nutrition	5	0.9	-0.0297	-0.0187	-0.1281	0.1094	1.08	0.2795
Exercise:Nutrition	3	0.78	0.1147	0.1406	0.0215	0.119	1.39	0.1651
Exercise+Nutrition:Nutrition	3	0.88	0.1444	0.1413	0.1661	-0.0247	-0.21	0.8319

Table S10: Side-splitting of Maximal gait speed. Inconsistency test between direct and indirect treatment comparisons in mixed treatment comparison.

comparison	k	prop	nma	direct	indir.	Diff	z	p-value
Exercise:CG	5	1	0.2075	0.2076	.	.	.	.
Exercise+Nutrition:CG	2	0.88	0.2168	0.21	0.2673	-0.0573	-0.23	0.8208
Nutrition:CG	2	0.87	0.0917	0.0859	0.1323	-0.0464	-0.19	0.8475
Exercise:Exercise+Nutrition	2	0.88	-0.0093	-0.0152	0.034	-0.0492	-0.19	0.8456
Exercise:Nutrition	2	0.88	0.1158	0.11	0.1573	-0.0473	-0.19	0.8455
Exercise+Nutrition:Nutrition	2	1	0.1251	0.1252	0.0709	0.0544	0.02	0.9826

Table S11: Side-splitting of ASM. Inconsistency test between direct and indirect treatment comparisons in mixed treatment comparison.

comparison	k	prop	nma	direct	indir.	Diff	z	p-value
Exercise:CG	10	0.99	0.1071	0.1145	-0.7555	0.87	1.28	0.2009
Exercise+Nutrition:CG	3	0.94	0.2145	0.1925	0.5875	-0.3949	-1.08	0.2822
Nutrition:CG	3	0.96	0.0935	0.0905	0.1729	-0.0823	-0.18	0.8549
Exercise:Exercise+Nutrition	4	0.97	-0.1073	-0.1137	0.0975	-0.2112	-0.43	0.6647
Exercise:Nutrition	3	0.95	0.0137	0.0149	-0.0102	0.0251	0.06	0.9489
Exercise+Nutrition:Nutrition	3	0.97	0.121	0.1045	0.7227	-0.6182	-1.01	0.3129

Table S12: Side-splitting of SMI. Inconsistency test between direct and indirect treatment comparisons in mixed treatment comparison.

comparison	k	prop	nma	direct	indir.	Diff	z	p-value
Exercise:CG	6	0.97	0.2186	0.2676	-1.4002	1.6678	1.68	0.0932
Exercise+Nutrition:CG	1	0.54	0.3232	-0.1284	0.8539	-0.9823	-1.82	0.0694
Nutrition:CG	1	0.79	0.2858	0.0642	1.0952	-1.031	-1.31	0.1912
Exercise:Exercise+Nutrition	2	0.89	-0.1046	-0.2169	0.8204	-1.0373	-1.31	0.1911
Exercise:Nutrition	1	0.76	-0.0672	-0.0642	-0.0764	0.0122	0.02	0.9868
Exercise+Nutrition:Nutrition	1	0.86	0.0374	-0.1926	1.4654	-1.6581	-1.68	0.1032

Section S4: Network forest plot

Figure S1: Network forest plot of Handgrip strength

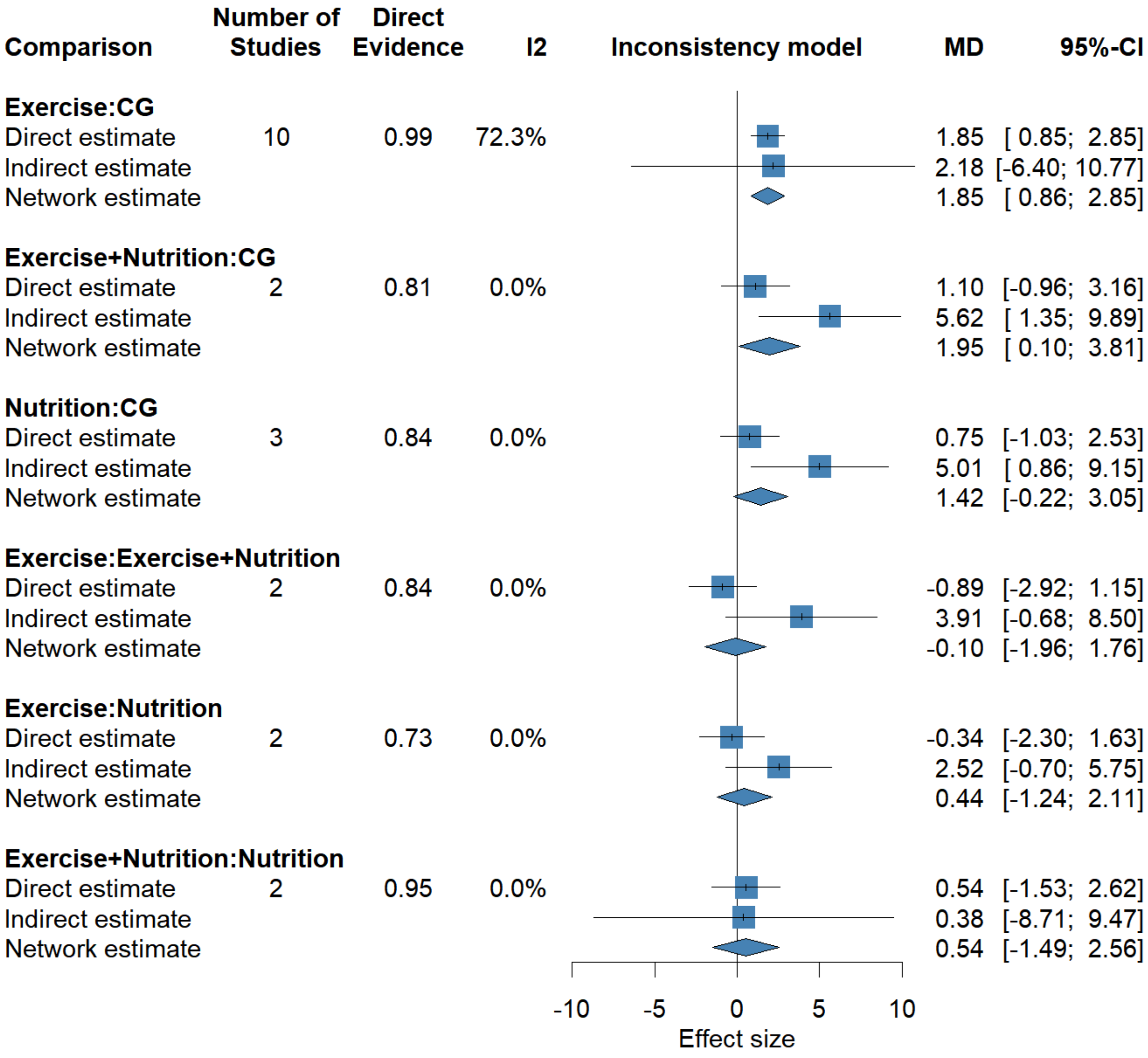

Figure S2: Network forest plot of Knee extension strength

Comparison	Number of Studies	Direct Evidence	I2	SMD	95%-CI
**Exercise:CG**					
Direct estimate	7	0.98	77.1%	0.75	[0.41; 1.09]
Indirect estimate				0.51	[-1.74; 2.75]
Network estimate				0.75	[0.41; 1.08]
**Exercise+Nutrition:CG**					
Direct estimate	3	0.80	0.0%	0.57	[0.09; 1.05]
Indirect estimate				1.28	[0.31; 2.26]
Network estimate				0.71	[0.28; 1.14]
**Nutrition:CG**					
Direct estimate	3	0.87	13.8%	0.21	[-0.27; 0.69]
Indirect estimate				1.22	[-0.03; 2.48]
Network estimate				0.34	[-0.11; 0.79]
**Exercise:Exercise+Nutrition**					
Direct estimate	4	0.90	0.0%	-0.07	[-0.51; 0.37]
Indirect estimate				0.96	[-0.32; 2.24]
Network estimate				0.04	[-0.37; 0.45]
**Exercise:Nutrition**					
Direct estimate	3	0.85	46.0%	0.29	[-0.19; 0.77]
Indirect estimate				1.08	[-0.06; 2.22]
Network estimate				0.41	[-0.04; 0.85]
**Exercise+Nutrition:Nutrition**					
Direct estimate	3	0.95	0.0%	0.36	[-0.12; 0.84]
Indirect estimate				0.58	[-1.62; 2.78]
Network estimate				0.37	[-0.10; 0.84]

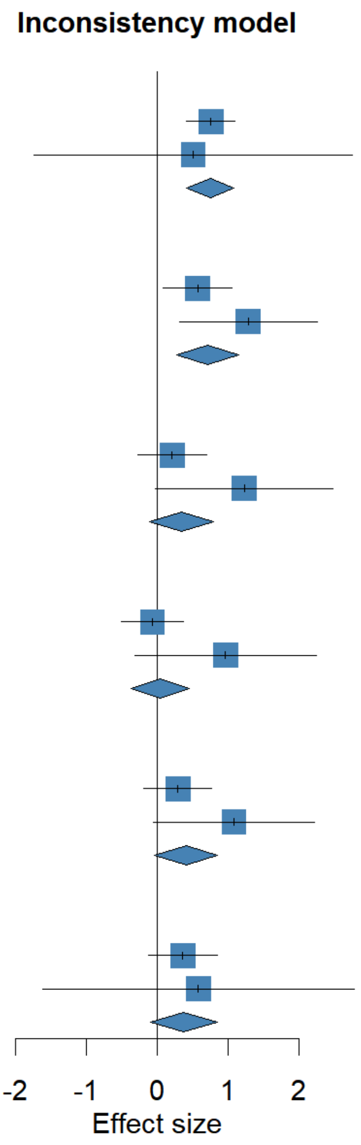

Figure S3: Network forest plot of Usual gait speed

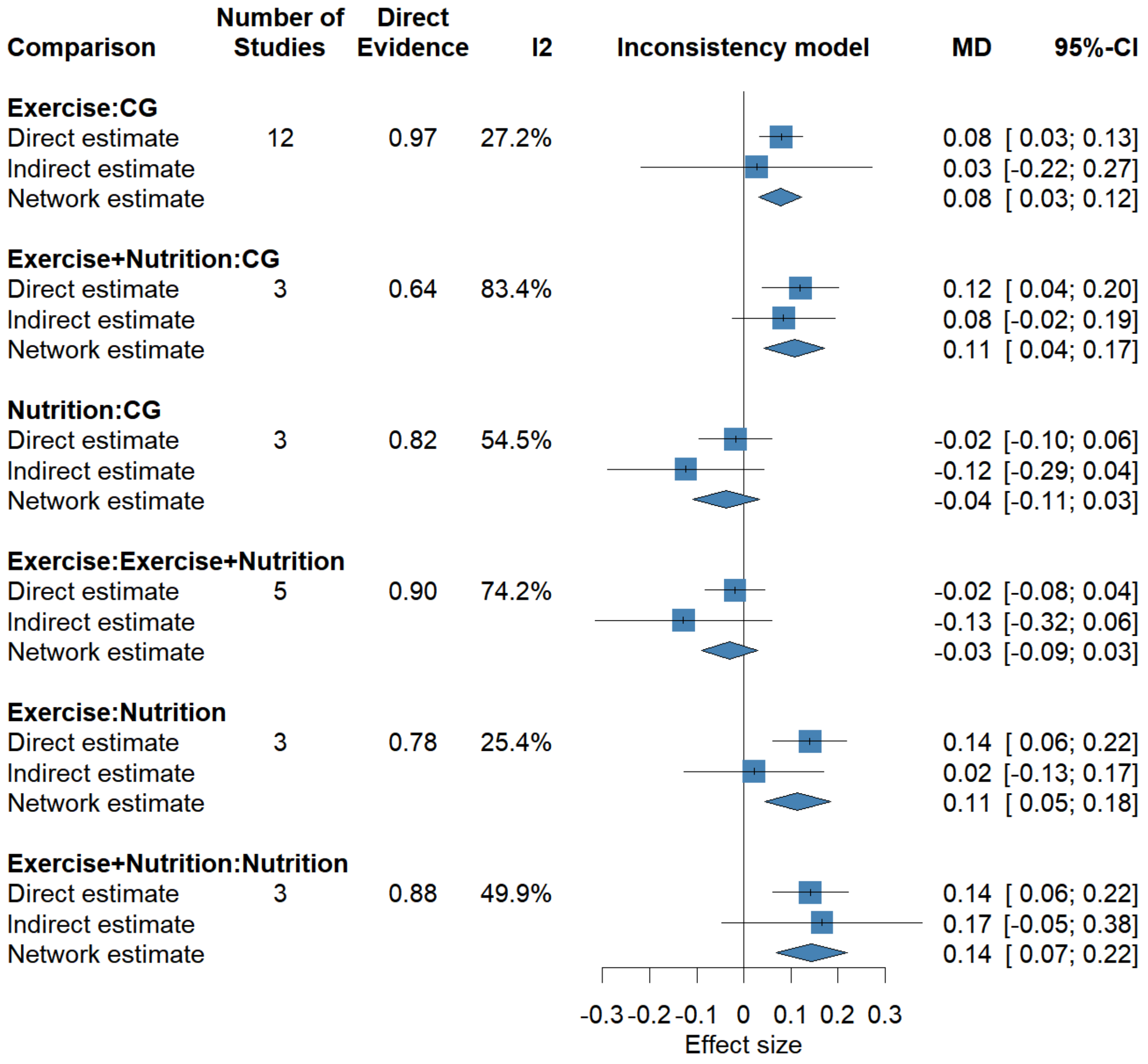

Figure S4: Network forest plot of Maximal gait speed

Comparison	Number of Studies	Direct Evidence	I2	MD	95%-CI
**Exercise+Nutrition:CG**					
Direct estimate	2	0.88	0.0%	0.21	[0.04; 0.38]
Indirect estimate				0.27	[-0.20; 0.73]
Network estimate				0.22	[0.06; 0.38]
**Nutrition:CG**					
Direct estimate	2	0.87	31.4%	0.09	[-0.08; 0.25]
Indirect estimate				0.13	[-0.31; 0.57]
Network estimate				0.09	[-0.06; 0.25]
**Exercise:Exercise+Nutrition**					
Direct estimate	2	0.88	17.0%	-0.02	[-0.19; 0.16]
Indirect estimate				0.03	[-0.43; 0.50]
Network estimate				-0.01	[-0.17; 0.15]
**Exercise:Nutrition**					
Direct estimate	2	0.88	0.0%	0.11	[-0.06; 0.28]
Indirect estimate				0.16	[-0.29; 0.60]
Network estimate				0.12	[-0.04; 0.27]
**Exercise+Nutrition:Nutrition**					
Direct estimate	2	1.00	16.8%	0.13	[-0.05; 0.30]
Indirect estimate				0.07	[-4.80; 4.94]
Network estimate				0.13	[-0.05; 0.30]

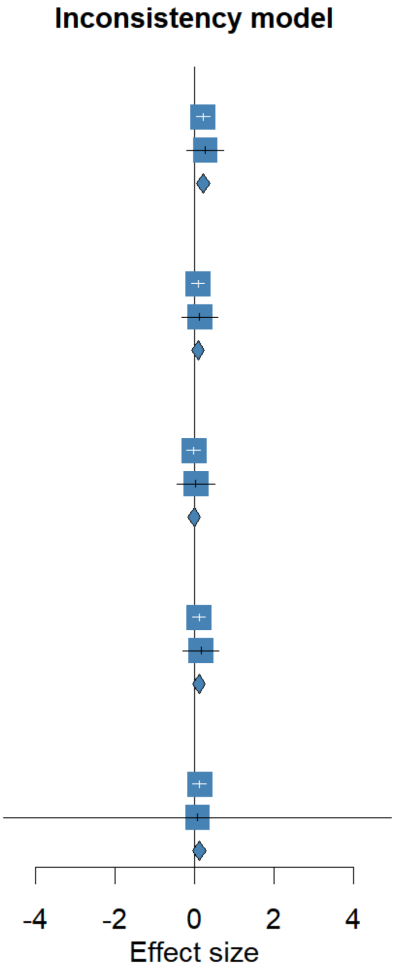

Figure S5: Network forest plot of ASM

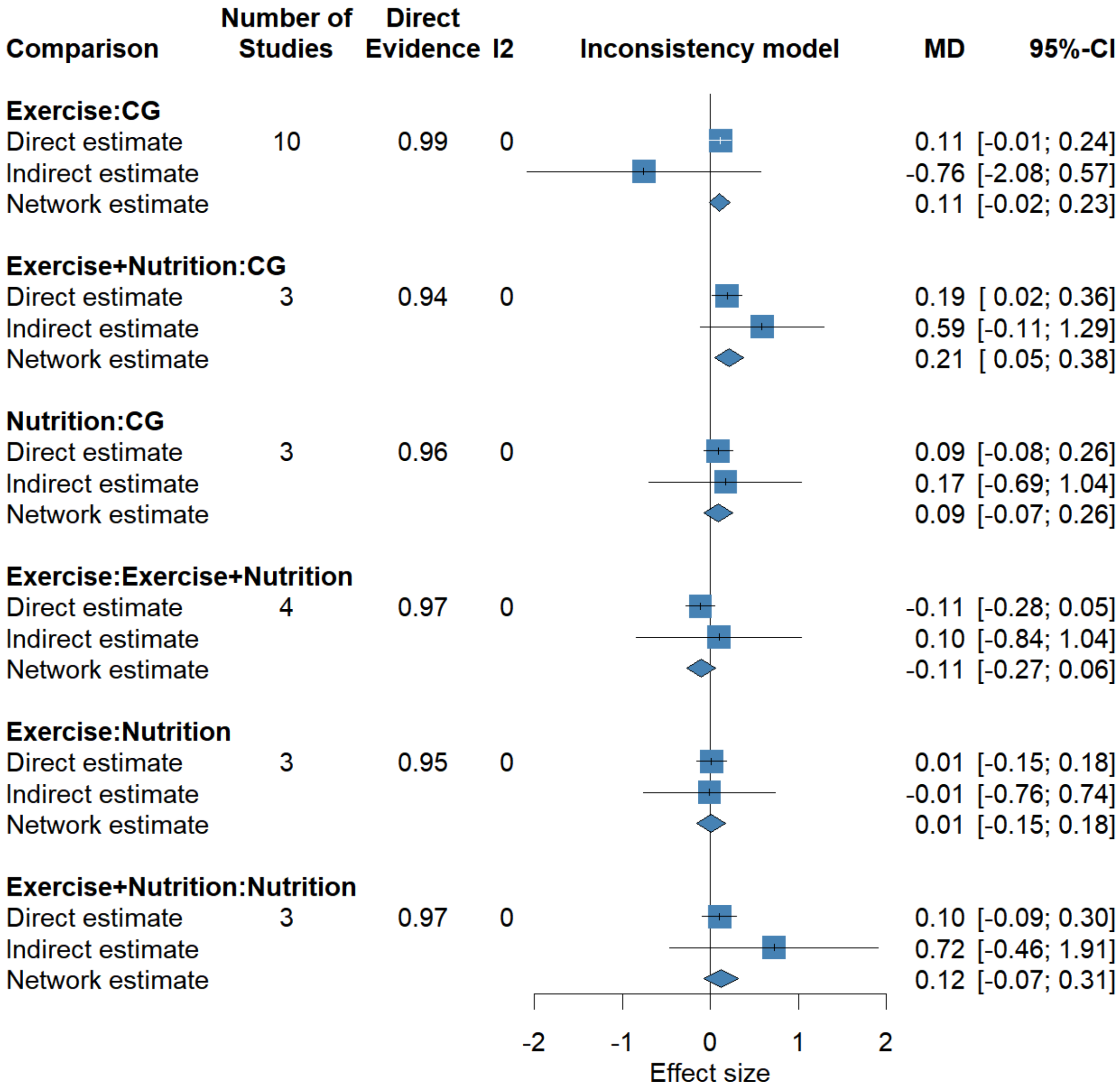

Figure S6: Network forest plot of SMI

Comparison	Number of Studies	Direct Evidence	I2	SMD	95%-CI
**Exercise:CG**					
Direct estimate	6	0.97	3.2%	0.27	[-0.07; 0.60]
Indirect estimate				-1.40	[-3.32; 0.52]
Network estimate				0.22	[-0.11; 0.55]
**Exercise+Nutrition:CG**					
Direct estimate	1	0.54		-0.13	[-0.85; 0.59]
Indirect estimate				0.85	[0.07; 1.63]
Network estimate				0.32	[-0.21; 0.85]
**Nutrition:CG**					
Direct estimate	1	0.79		0.06	[-0.65; 0.78]
Indirect estimate				1.10	[-0.27; 2.47]
Network estimate				0.29	[-0.35; 0.92]
**Exercise:Exercise+Nutrition**					
Direct estimate	2	0.89	77.2%	-0.22	[-0.73; 0.29]
Indirect estimate				0.82	[-0.65; 2.29]
Network estimate				-0.10	[-0.59; 0.38]
**Exercise:Nutrition**					
Direct estimate	1	0.76		-0.06	[-0.78; 0.65]
Indirect estimate				-0.08	[-1.34; 1.18]
Network estimate				-0.07	[-0.69; 0.56]
**Exercise+Nutrition:Nutrition**					
Direct estimate	1	0.86		-0.19	[-0.91; 0.53]
Indirect estimate				1.47	[-0.33; 3.26]
Network estimate				0.04	[-0.63; 0.71]

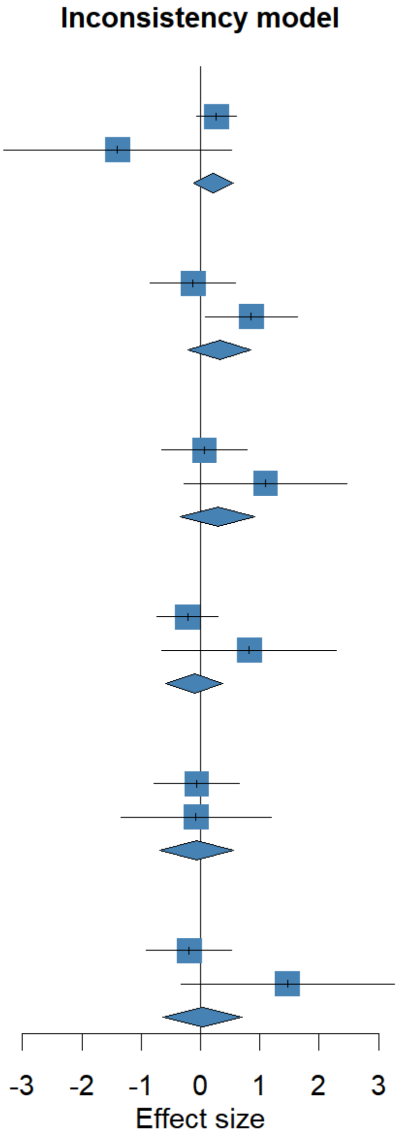

Section S5: League table

Table S13: league table of Handgrip strength

This table presents both direct and network comparisons of intervention effects on Gait speed. All effect estimates are expressed as MD with 95% CI. Results from the network meta-analysis are displayed in the lower triangle, while pairwise meta-analysis results are shown in the upper triangle. Statistically significant results are presented in bold. The shading color indicates the confidence rating of the evidence, as assessed using the CINeMA framework: blue denotes high confidence, green moderate, orange low, and red very low.

Low certainty	Very low certainty

Exercise	-0.89 (-2.92; 1.15)	-0.34 (-2.30; 1.63)	**1.85 (0.85; 2.85)**
-0.10 (-1.96; 1.76)	Exercise+Nutrition	0.54 (-1.53; 2.62)	1.10 (-0.96; 3.16)
0.44 (-1.24; 2.11)	0.54 (-1.49; 2.56)	Nutrition	0.75 (-1.03; 2.53)
**1.85 (0.86; 2.85)**	**1.95 (0.10; 3.81)**	1.42 (-0.22; 3.05)	CG

Table S14: league table of Knee extension strength

This table presents both direct and network comparisons of intervention effects on Knee extensor strength. All effect estimates are expressed as SMD with 95% CI. Results from the network meta-analysis are displayed in the lower triangle, while pairwise meta-analysis results are shown in the upper triangle. Statistically significant results are presented in bold. The shading color indicates the confidence rating of the evidence, as assessed using the CINeMA framework: blue denotes high confidence, green moderate, orange low, and red very low.

Low certainty	Very low certainty

Exercise	-0.07 (-0.51; 0.37)	0.29 (-0.19; 0.77)	**0.75 (0.41; 1.09)**
0.04 (-0.37; 0.45)	Exercise+Nutrition	0.36 (-0.12; 0.84)	**0.57 (0.09; 1.05)**
0.41 (-0.04; 0.85)	0.37 (-0.10; 0.84)	Nutrition	0.21 (-0.27; 0.69)
**0.75 (0.41; 1.08)**	**0.71 (0.28; 1.14)**	0.34 (-0.11; 0.79)	CG

Table S15: league table of Usual gait speed

This table presents both direct and network comparisons of intervention effects on Five repetition chair stand test. All effect estimates are expressed as MD with 95% CI. Results from the network meta-analysis are displayed in the lower triangle, while pairwise meta-analysis results are shown in the upper triangle. Statistically significant results are presented in bold. The shading color indicates the confidence rating of the evidence, as assessed using the CINeMA framework: blue denotes high confidence, green moderate, orange low, and red very low.

Moderate certainty	Low certainty	Very low certainty

Exercise	-0.02 (-0.08; 0.04)	**0.14 (0.06; 0.22)**	**0.08 (0.03; 0.13)**
-0.03 (-0.09; 0.03)	Exercise+Nutrition	**0.14 (0.06; 0.22)**	**0.12 (0.04; 0.20)**
**0.11 (0.05; 0.18)**	**0.14 (0.07; 0.22)**	Nutrition	-0.02 (-0.10; 0.06)
**0.08 (0.03; 0.12)**	**0.11 (0.04; 0.17)**	-0.04 (-0.11; 0.03)	CG

Table S16: league table of Maximal gait speed

This table presents both direct and network comparisons of intervention effects on Timed up and go. All effect estimates are expressed as MD with 95% CI. Results from the network meta-analysis are displayed in the lower triangle, while pairwise meta-analysis results are shown in the upper triangle. Statistically significant results are presented in bold. The shading color indicates the confidence rating of the evidence, as assessed using the CINeMA framework: blue denotes high confidence, green moderate, orange low, and red very low.

Low certainty

Exercise	-0.02 (-0.19; 0.16)	0.11 (-0.06; 0.28)	**0.21 (0.09; 0.33)**
-0.01 (-0.17; 0.15)	Exercise+Nutrition	0.13 (-0.05; 0.30)	**0.21 (0.04; 0.38)**
0.12 (-0.04; 0.27)	0.13 (-0.05; 0.30)	Nutrition	0.09 (-0.08; 0.25)
**0.21 (0.09; 0.33)**	**0.22 (0.06; 0.38)**	0.09 (-0.06; 0.25)	CG

Table S17: league table of ASM

This table presents both direct and network comparisons of intervention effects on Balance test. All effect estimates are expressed as SMD with 95% CI. Results from the network meta-analysis are displayed in the lower triangle, while pairwise meta-analysis results are shown in the upper triangle. Statistically significant results are presented in bold. The shading color indicates the confidence rating of the evidence, as assessed using the CINeMA framework: blue denotes high confidence, green moderate, orange low, and red very low.

High certainty	Low certainty

Exercise	-0.11 (-0.28; 0.05)	0.01 (-0.15; 0.18)	0.11 (-0.01; 0.24)
-0.11 (-0.27; 0.06)	Exercise+Nutrition	0.10 (-0.09; 0.30)	**0.19 (0.02; 0.36)**
0.01 (-0.15; 0.18)	0.12 (-0.07; 0.31)	Nutrition	0.09 (-0.08; 0.26)
0.11 (-0.02; 0.23)	**0.21 (0.05; 0.38)**	0.09 (-0.07; 0.26)	CG

Table S18: league table of SMI

This table presents both direct and network comparisons of intervention effects on Balance test. All effect estimates are expressed as MD with 95% CI. Results from the network meta-analysis are displayed in the lower triangle, while pairwise meta-analysis results are shown in the upper triangle. Statistically significant results are presented in bold. The shading color indicates the confidence rating of the evidence, as assessed using the CINeMA framework: blue denotes high confidence, green moderate, orange low, and red very low.

Low certainty

Exercise	-0.22 (-0.73; 0.29)	-0.06 (-0.78; 0.65)	0.27 (-0.07; 0.60)
-0.10 (-0.59; 0.38)	Exercise+Nutrition	-0.19 (-0.91; 0.53)	-0.13 (-0.85; 0.59)
-0.07 (-0.69; 0.56)	0.04 (-0.63; 0.71)	Nutrition	0.06 (-0.65; 0.78)
0.22 (-0.11; 0.55)	0.32 (-0.21; 0.85)	0.29 (-0.35; 0.92)	CG

Section S6: CINeMA Assessment

We use the CINeMA framework to assess evidence certainty, evaluating each network estimate based on the following criteria:

- **Within study bias:** We classified the overall risk of bias for each study as low risk of bias, the risk of bias as moderate when none of the four assessed risk of bias items were rated as high risk, and the risk of bias as high when one or both items were rated as high risk. See **Appendix 2** for the bias assessment.
- **Reporting bias:** We judged it visually by a funnel plot **(Appendix 3)**.
- **Indirectness:** Transferability assumptions were assessed by reporting baseline glycated hemoglobin levels in the included study population and by comparing age and BMI at baseline concordance between groups.
- **Imprecision:** We use the CINeMA website to grade the accuracy of each comparison.
- **Heterogeneity:** We assessed the degree of worry by comparing clinical reasoning based on 95% confidence intervals (CIs) while applying the same clinical reasoning framework as for inaccuracy. In particular, we judged the consistency of our findings based on the confidence and prediction intervals associated with clinically important effect sizes. And we used the same thresholds of clinical significance as described above and followed the recommendations automatically provided by CINeMA (https://cinema.ispm.unibe.ch/).
- **Inconsistency:** For inconsistency, we looked at the results for node splitting **(Appendix 3)** and we saw major problems when p<0.10, but otherwise no problems.

Figure S7: Risk of bias contribution by intervention group in Handgrip strength

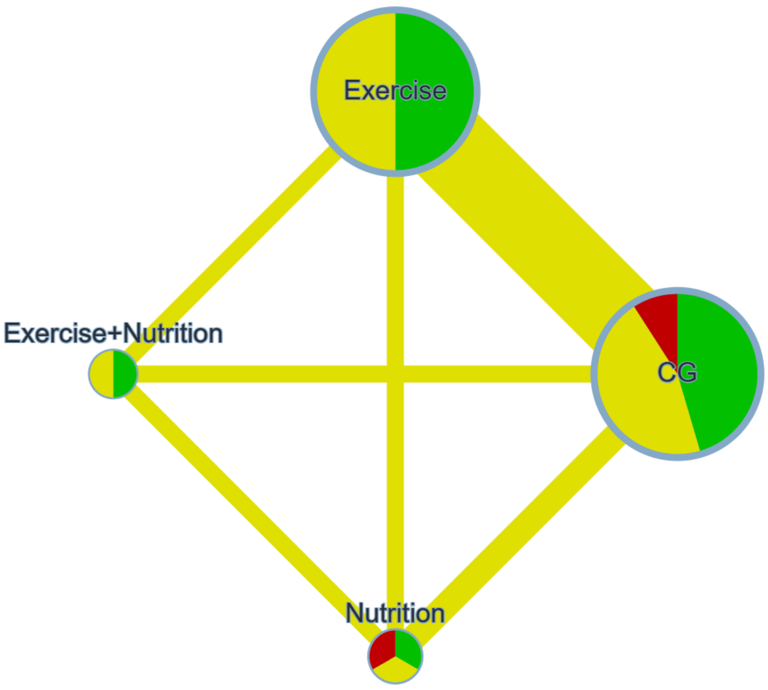

Table S19: CINeMA Results of Handgrip strength

Comparison	Within-study bias	Reporting bias	Indirectness	Imprecision	Heterogeneity	Incoherence	Confidence rating
CG:Exercise	Some concerns	Low risk	No concerns	No concerns	Major concerns	No concerns	Very low
CG:Exercise+ Nutrition	No concerns	Low risk	No concerns	No concerns	Major concerns	No concerns	Low
CG:Nutrition	No concerns	Low risk	No concerns	Major concerns	No concerns	No concerns	Low
Exercise:Exercise+Nutrition	No concerns	Low risk	No concerns	Major concerns	No concerns	No concerns	Low
Exercise:Nutrition	No concerns	Low risk	No concerns	Major concerns	No concerns	No concerns	Low
Exercise+Nutrition:Nutrition	No concerns	Low risk	No concerns	Major concerns	No concerns	No concerns	Low

Figure S8: Risk of bias contribution by intervention group in Knee extension strength

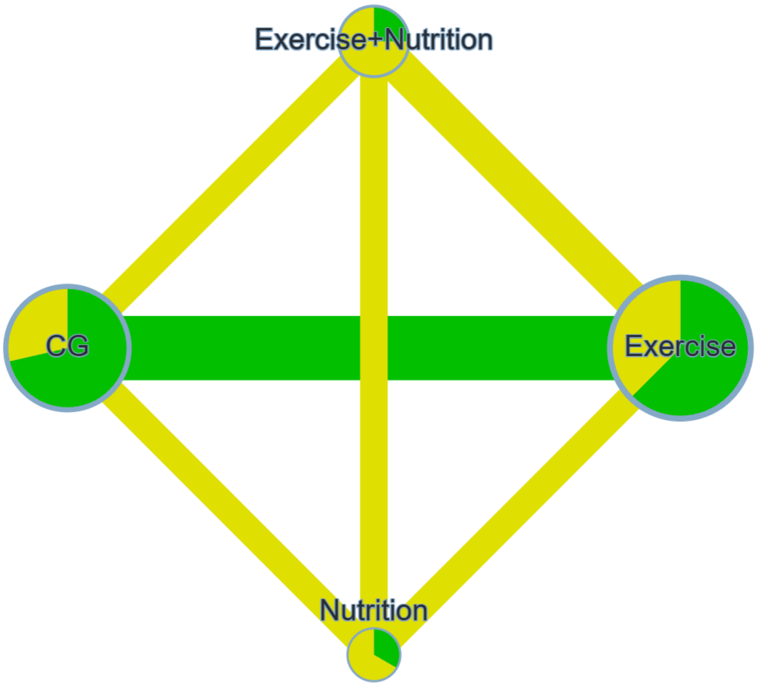

Table S20: CINeMA Results of Knee extension strength

Comparison	Within-study bias	Reporting bias	Indirectness	Imprecision	Heterogeneity	Incoherence	Confidence rating
CG:Exercise	No concerns	Low risk	No concerns	No concerns	Major concerns	No concerns	Low
CG:Exercise+ Nutrition	Some concerns	Low risk	No concerns	No concerns	Major concerns	No concerns	Very low
CG:Nutrition	Some concerns	Low risk	No concerns	Major concerns	No concerns	No concerns	Very low
Exercise:Exercise+Nutrition	Some concerns	Low risk	No concerns	Major concerns	No concerns	No concerns	Very low
Exercise:Nutrition	Some concerns	Low risk	No concerns	Major concerns	No concerns	No concerns	Very low
Exercise+Nutrition:Nutrition	Some concerns	Low risk	No concerns	Major concerns	No concerns	No concerns	Very low
CG:Exercise	No concerns	Low risk	No concerns	No concerns	Major concerns	No concerns	Low

Figure S9: Risk of bias contribution by intervention group in Usual gait speed

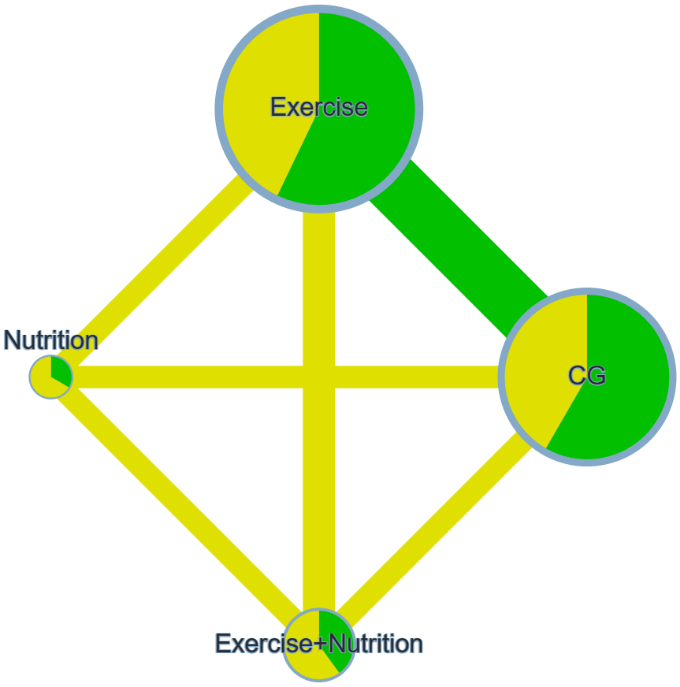

Table S21: CINeMA Results of Usual gait speed

Comparison	Within-study bias	Reporting bias	Indirectness	Imprecision	Heterogeneity	Incoherence	Confidence rating
CG:Exercise	No concerns	Low risk	No concerns	No concerns	Major concerns	No concerns	Low
CG:Exercise +Nutrition	Some concerns	Low risk	No concerns	No concerns	Major concerns	No concerns	Very low
CG:Nutrition	Some concerns	Low risk	No concerns	Major concerns	No concerns	No concerns	Very low
Exercise:Exercise+Nutrition	Some concerns	Low risk	No concerns	Major concerns	No concerns	No concerns	Very low
Exercise:Nutrition	Some concerns	Low risk	No concerns	No concerns	Major concerns	No concerns	Very low
Exercise+Nutrition:Nutrition	Some concerns	Low risk	No concerns	No concerns	No concerns	No concerns	Moderate

Figure S10: Risk of bias contribution by intervention group in Maximal gait speed

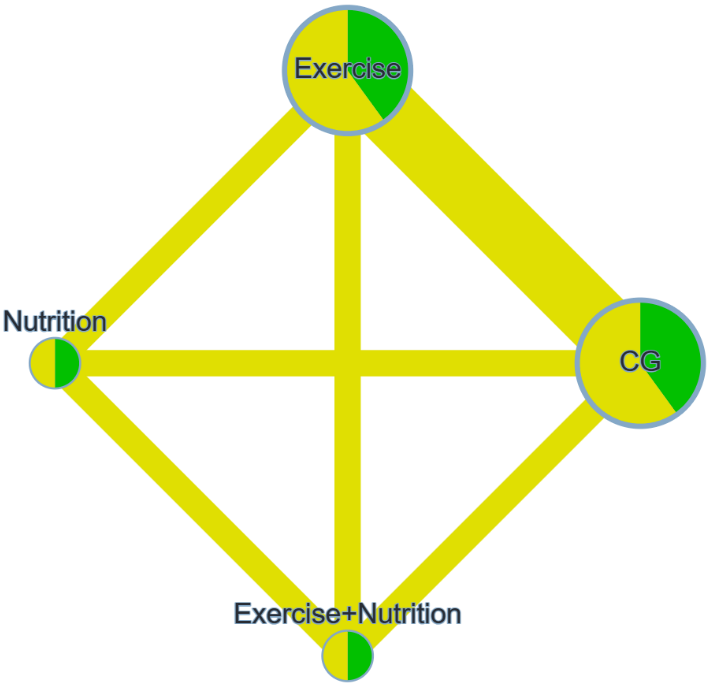

Table S22: CINeMA Results of Maximal gait speed

Comparison	Within-study bias	Reporting bias	Indirectness	Imprecision	Heterogeneity	Incoherence	Confidence rating
CG:Exercise	No concerns	Low risk	No concerns	No concerns	Major concerns	No concerns	Low
CG:Exercise+Nutrition	No concerns	Low risk	No concerns	No concerns	Major concerns	No concerns	Low
CG:Nutrition	No concerns	Low risk	No concerns	Major concerns	No concerns	No concerns	Low
Exercise:Exercise+Nutrition	No concerns	Low risk	No concerns	Major concerns	No concerns	No concerns	Low
Exercise:Nutrition	No concerns	Low risk	No concerns	Major concerns	No concerns	No concerns	Low
Exercise+Nutrition:Nutrition	No concerns	Low risk	No concerns	Major concerns	No concerns	No concerns	Low

Figure S11: Risk of bias contribution by intervention group in ASM

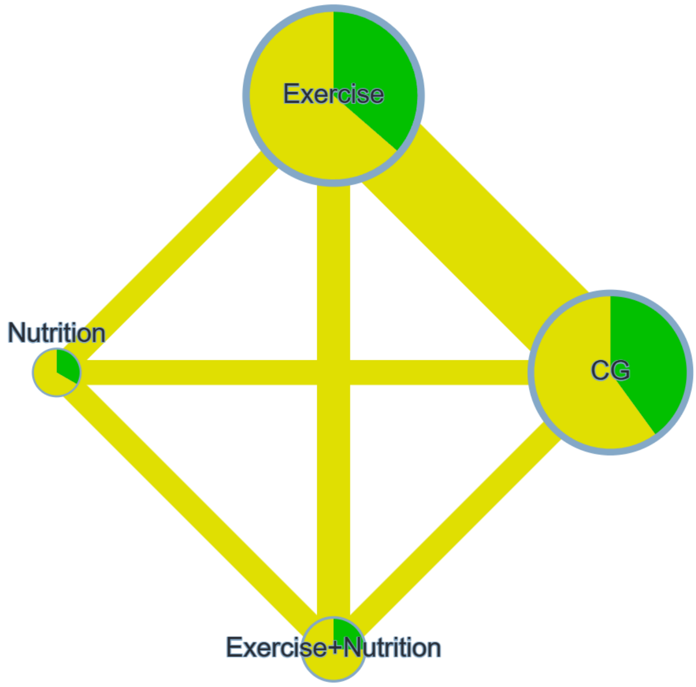

Table S23: CINeMA Results of ASM

Comparison	Within-study bias	Reporting bias	Indirectness	Imprecision	Heterogeneity	Incoherence	Confidence rating
CG:Exercise	No concerns	Low risk	No concerns	Major concerns	No concerns	No concerns	Low
CG:Exercise+Nutrition	No concerns	Low risk	No concerns	No concerns	No concerns	No concerns	High
CG:Nutrition	No concerns	Low risk	No concerns	Major concerns	No concerns	No concerns	Low
Exercise:Exercise+Nutrition	No concerns	Low risk	No concerns	Major concerns	No concerns	No concerns	Low
Exercise:Nutrition	No concerns	Low risk	No concerns	Major concerns	No concerns	No concerns	Low
Exercise+Nutrition:Nutrition	No concerns	Low risk	No concerns	Major concerns	No concerns	No concerns	Low

Figure S12: Risk of bias contribution by intervention group in SMI

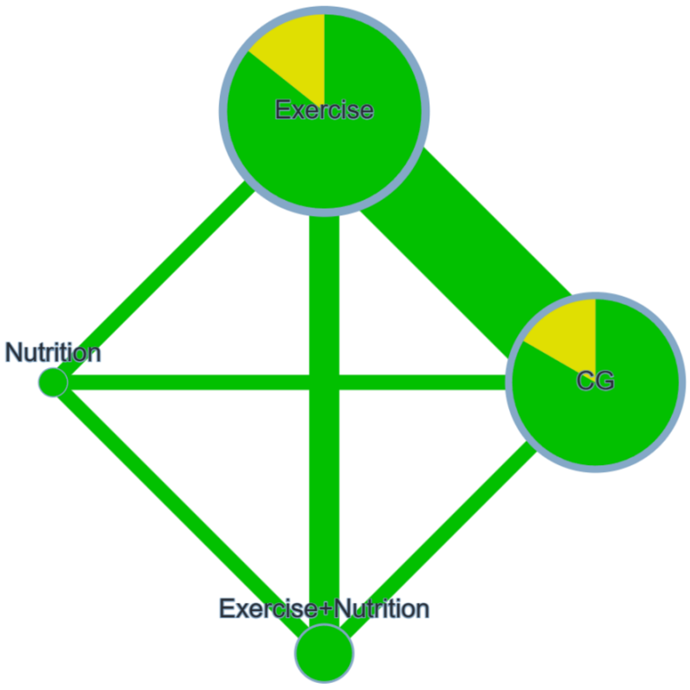

Table S24: CINeMA Results of SMI

Comparison	Within-study bias	Reporting bias	Indirectness	Imprecision	Heterogeneity	Incoherence	Confidence rating
CG:Exercise	No concerns	Low risk	No concerns	Major concerns	No concerns	No concerns	Low
CG:Exercise +Nutrition	No concerns	Low risk	No concerns	Major concerns	No concerns	No concerns	Low
CG:Nutrition	No concerns	Low risk	No concerns	Major concerns	No concerns	No concerns	Low
Exercise:Exercise+Nutrition	No concerns	Low risk	No concerns	Major concerns	No concerns	No concerns	Low
Exercise:Nutrition	No concerns	Low risk	No concerns	Major concerns	No concerns	No concerns	Low
Exercise+Nutrition:Nutrition	No concerns	Low risk	No concerns	Major concerns	No concerns	No concerns	Low

Section S7: Funnel Plots with Egger's Test for Publication Bias

Figure S13: Funnel plot of Handgrip strength

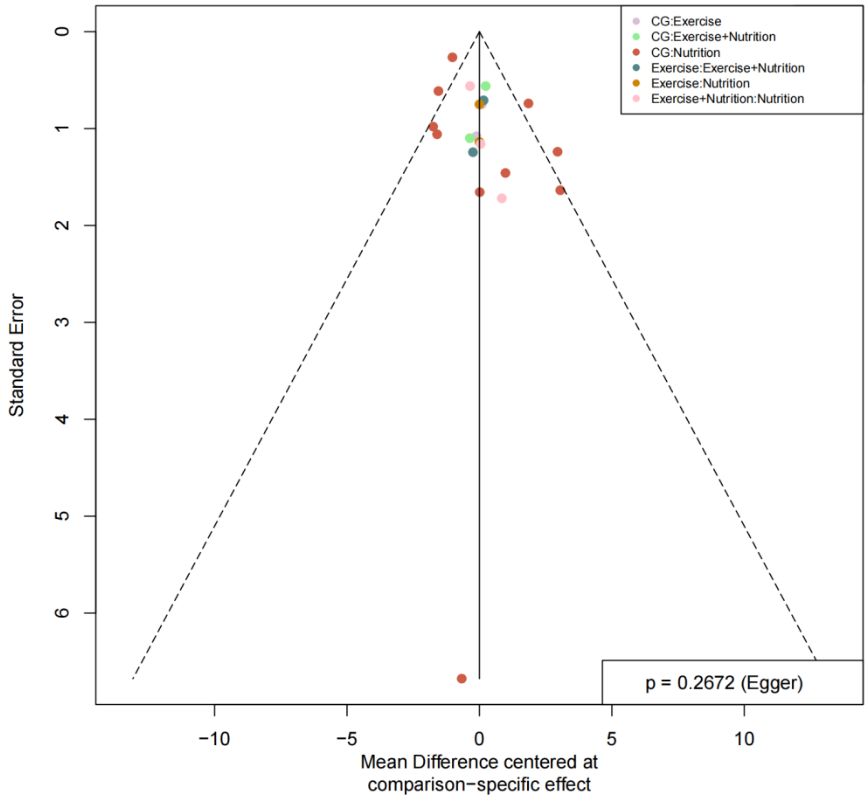

Figure S14: Funnel plot of Knee extension strength

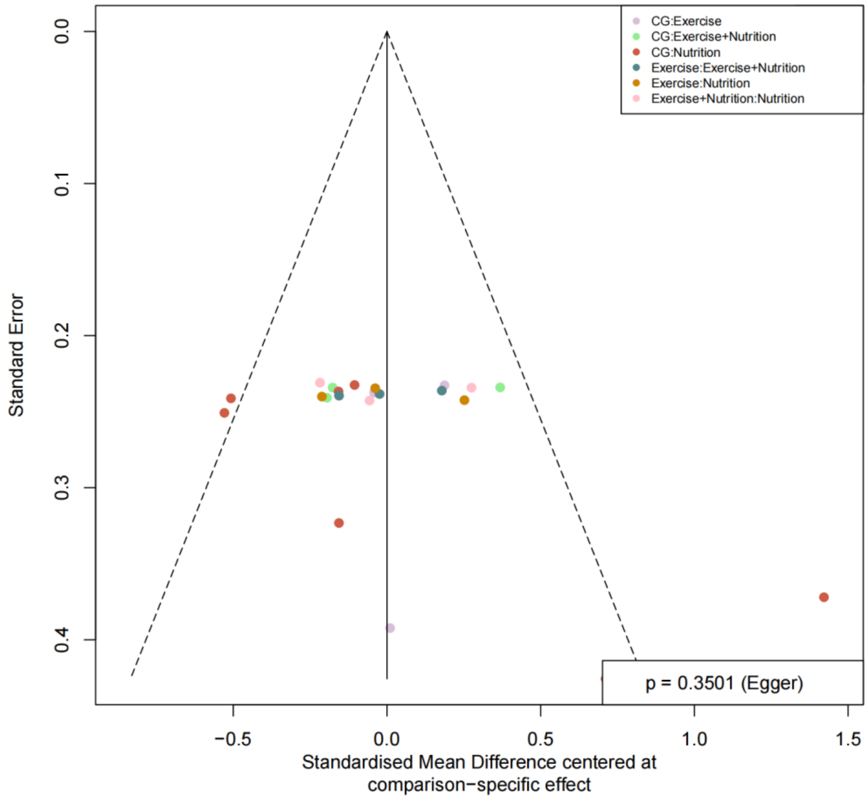

Figure S15: Funnel plot of Usual gait speed

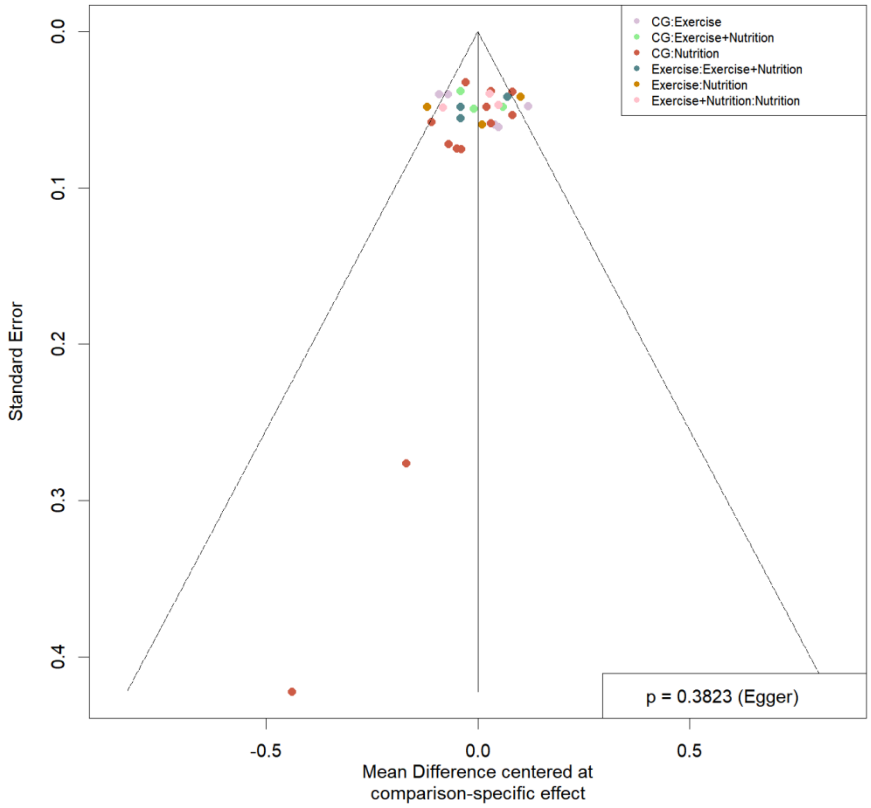

Figure S16: Funnel plot of Maximal gait speed

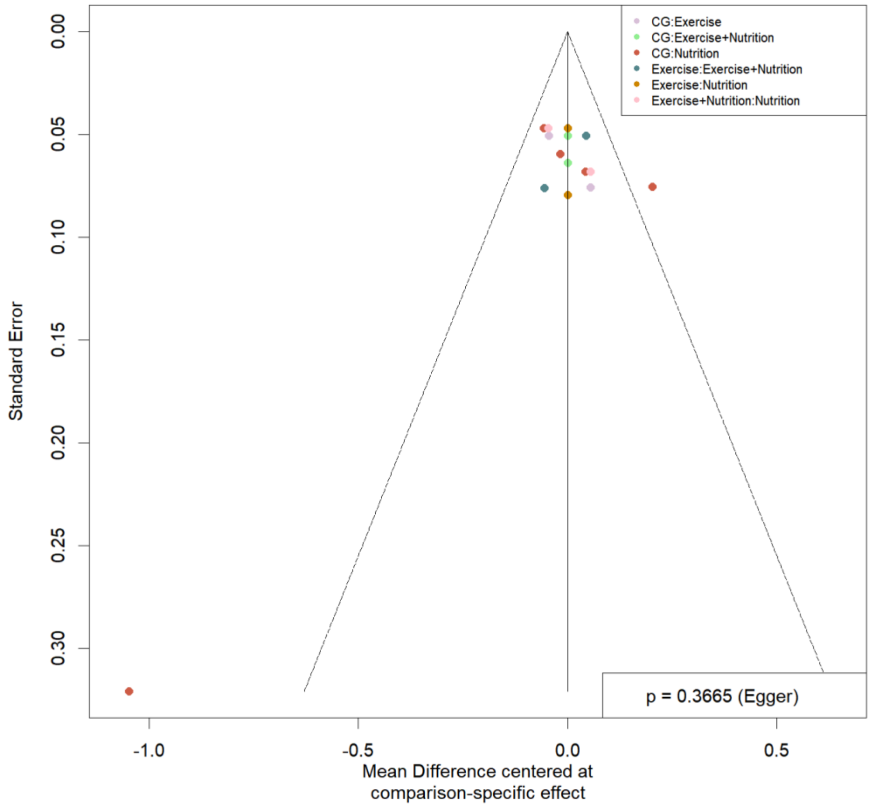

Figure S17: Funnel plot of ASM

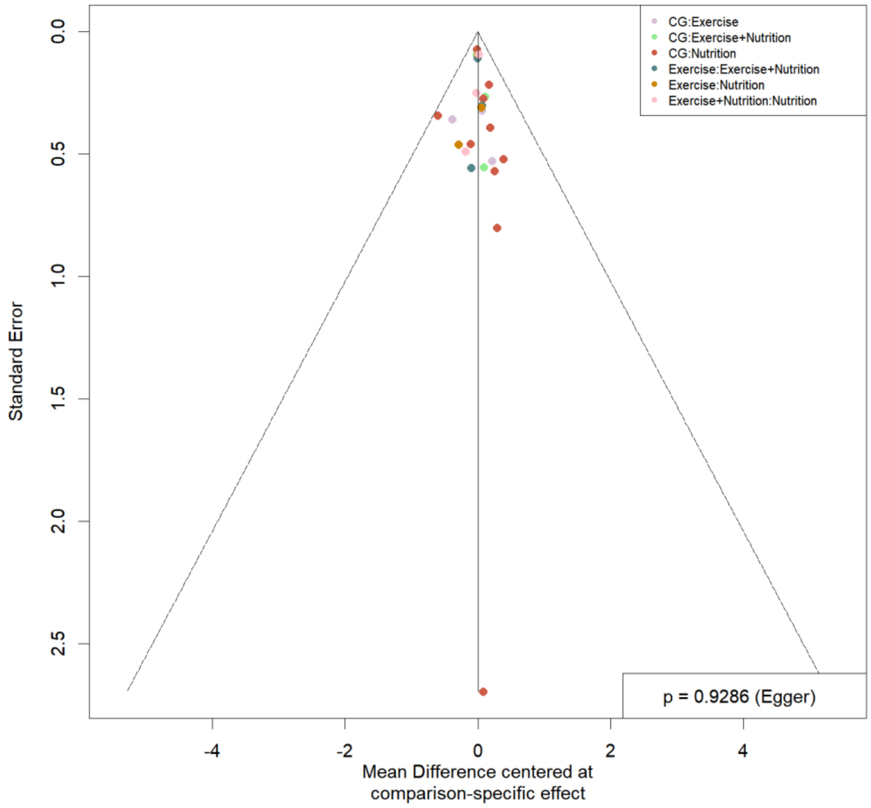

Figure S18: Funnel plot of SMI

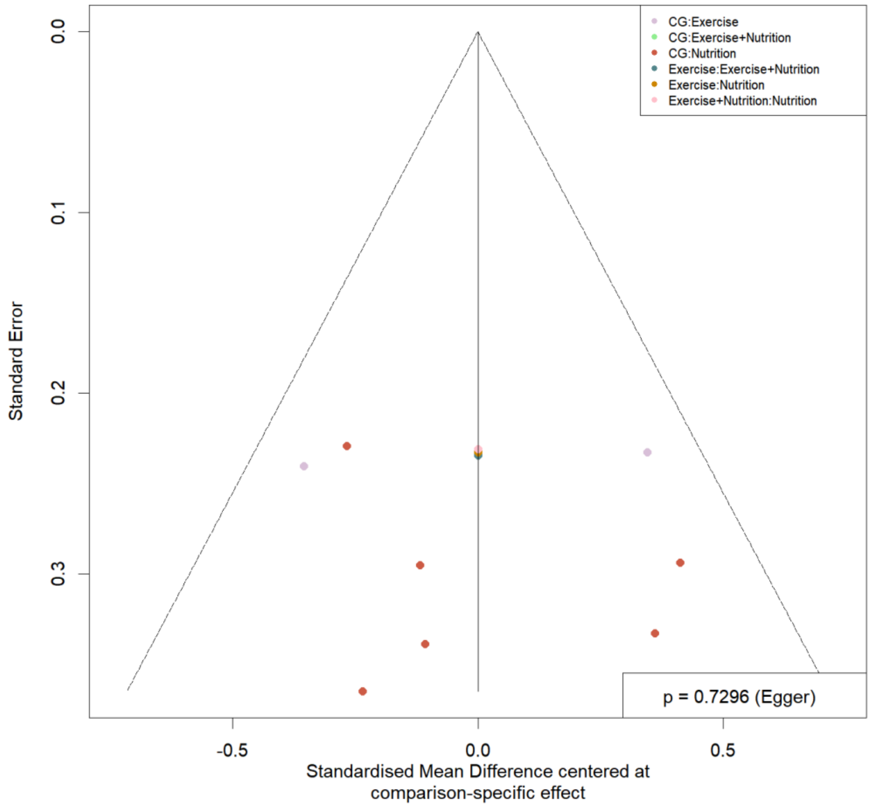

Section S8: Subgroup Analysis of Different Exercise Modalities

Figure S19 Network map of Handgrip Strength

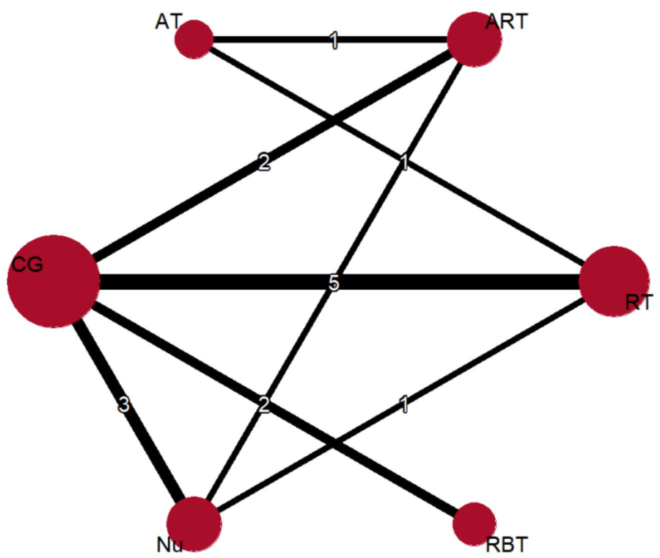

Figure S20 Forest plot of Handgrip Strength

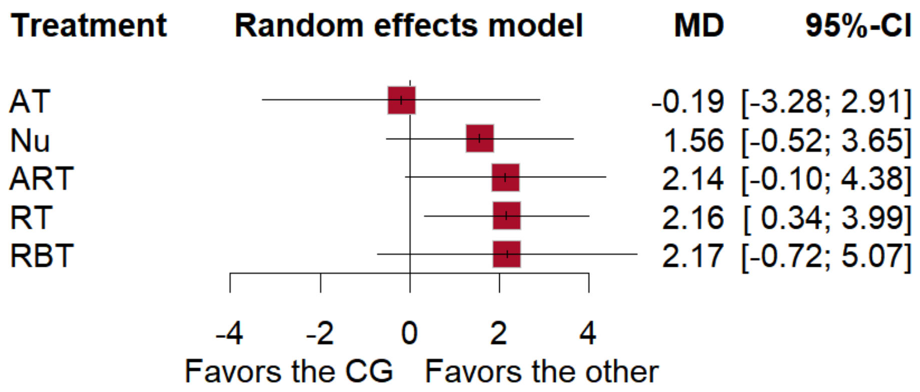

Table S25 League table of Handgrip strength

ART	2.80 (-0.98; 6.58)	-0.70 (-4.53; 3.13)	.	.	2.01 (-0.53; 4.55)
2.33 (-0.65; 5.30)	AT	.	.	-1.88 (-5.65; 1.89)	.
0.58 (-2.11; 3.27)	-1.75 (-5.20; 1.70)	Nu	.	0.10 (-3.25; 3.45)	0.81 (-1.42; 3.03)
-0.04 (-3.70; 3.63)	-2.36 (-6.60; 1.88)	-0.61 (-4.18; 2.96)	RBT	.	2.17 (-0.72; 5.07)
-0.03 (-2.65; 2.60)	-2.35 (-5.32; 0.62)	-0.60 (-3.02; 1.82)	0.01 (-3.42; 3.43)	RT	**2.24 (0.28; 4.20)**
2.14 (-0.10; 4.38)	-0.19 (-3.28; 2.91)	1.56 (-0.52; 3.65)	2.17 (-0.72; 5.07)	**2.16 (0.34; 3.99)**	CG

Figure S21 Network map of Knee extension strength

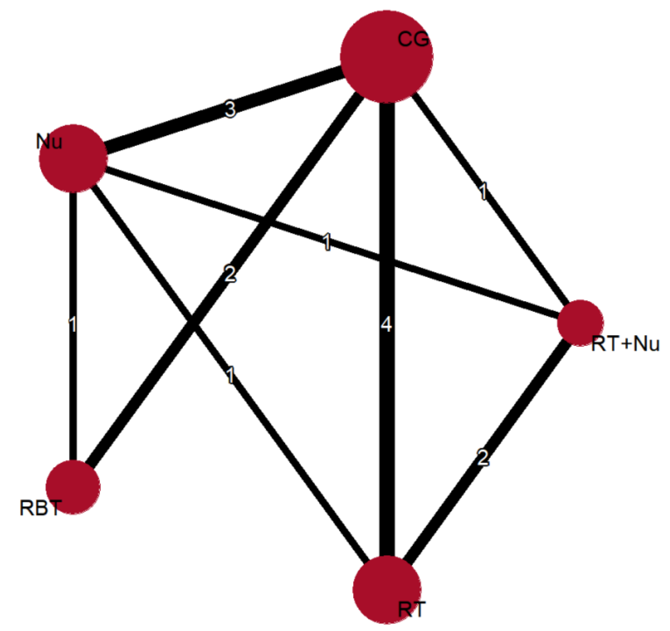

Figure S22 Forest plot of Knee extension strength

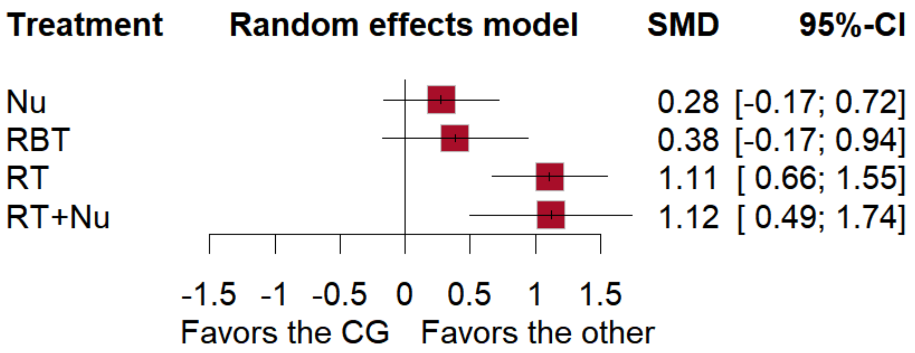

Table S26 League table of Knee extension strength

Nu	-0.11 (-0.93; 0.70)	-0.66 (-1.47; 0.16)	-0.76 (-1.58; 0.05)	0.21 (-0.26; 0.68)
-0.11 (-0.73; 0.52)	RBT	.	.	0.42 (-0.16; 1.00)
-0.83 (-1.39; -0.27)	-0.72 (-1.42; -0.03)	RT	-0.17 (-0.81; 0.47)	**1.13 (0.66; 1.60)**
-0.84 (-1.52; -0.17)	-0.74 (-1.55; 0.08)	-0.01 (-0.61; 0.59)	RT+Nu	0.76 (-0.06; 1.57)
0.28 (-0.17; 0.72)	0.38 (-0.17; 0.94)	**1.11 (0.66; 1.55)**	**1.12 (0.49; 1.74)**	CG

Figure S23 Network map of Usual gait speed

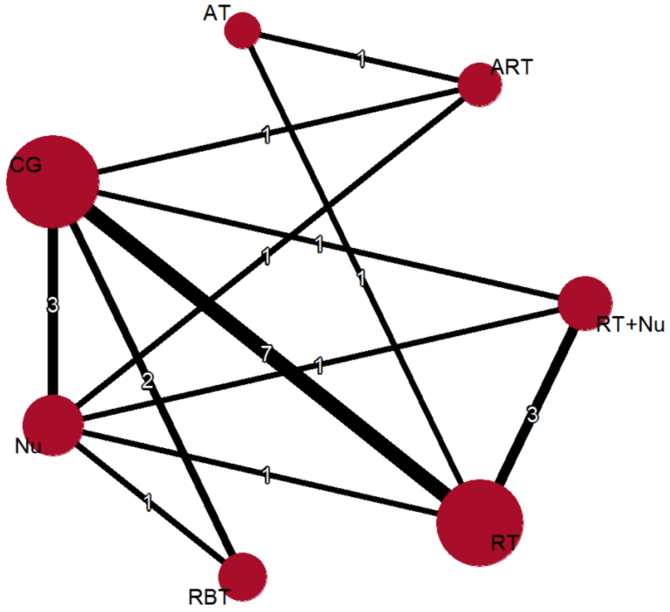

Figure S24 Forest plot of Usual gait speed

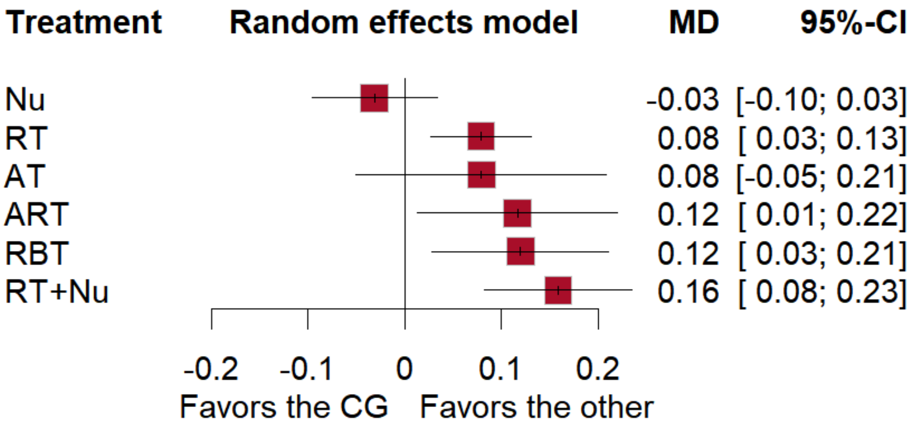

Table S27 League table of Usual gait speed

ART	0.02 (-0.09; 0.13)	**0.20 (0.07; 0.33)**	.	.	.	0.10 (-0.03; 0.23)
0.04 (-0.06; 0.14)	AT	.	.	-0.07 (-0.29; 0.15)	.	.
**0.15 (0.04; 0.25)**	0.11 (-0.02; 0.24)	Nu	-0.13 (-0.26; 0.00)	-0.10 (-0.21; 0.01)	**-0.21 (-0.33; -0.09)**	-0.02 (-0.09; 0.05)
-0.00 (-0.14; 0.13)	-0.04 (-0.20; 0.11)	**-0.15 (-0.25; -0.05)**	RBT	.	.	**0.14 (0.04; 0.23)**
0.04 (-0.07; 0.15)	0.00 (-0.13; 0.13)	**-0.11 (-0.18; -0.04)**	0.04 (-0.06; 0.14)	RT	-0.07 (-0.14; 0.00)	**0.07 (0.01; 0.13)**
-0.04 (-0.16; 0.08)	-0.08 (-0.22; 0.06)	**-0.19 (-0.28; -0.10)**	-0.04 (-0.15; 0.08)	**-0.08 (-0.15; -0.01)**	RT+Nu	**0.22 (0.10; 0.34)**
**0.12 (0.01; 0.22)**	0.08 (-0.05; 0.21)	-0.03 (-0.10; 0.03)	**0.12 (0.03; 0.21)**	**0.08 (0.03; 0.13)**	**0.16 (0.08; 0.23)**	CG

Figure S25 Network map of Maximal gait speed

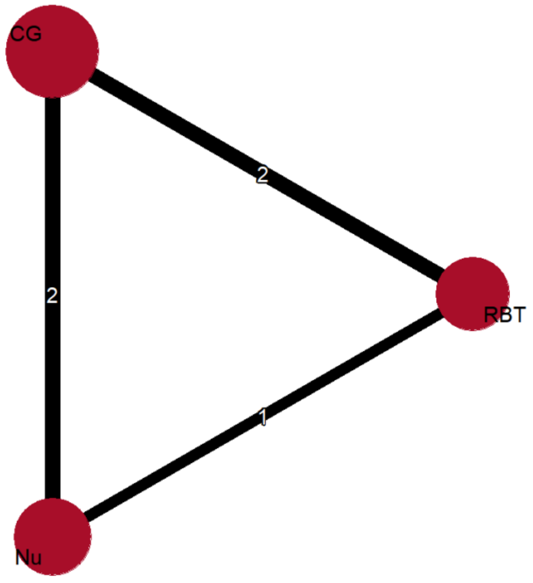

Figure S26 Forest plot of Maximal gait speed

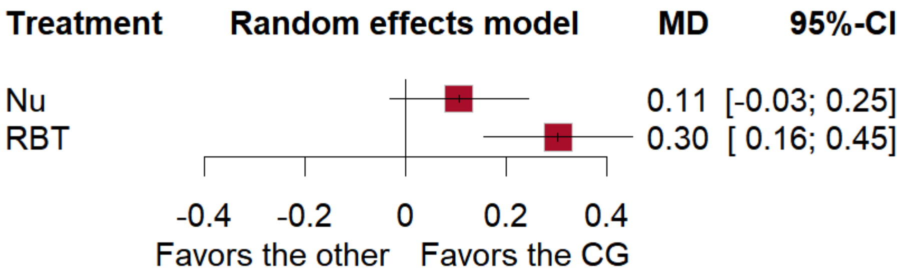

Table S28 League table of Maximal gait speed

Nu	-0.11 (-0.32; 0.10)	0.08 (-0.06; 0.23)
**-0.20 (-0.37; -0.03)**	RBT	**0.33 (0.17; 0.48)**
0.11 (-0.03; 0.25)	**0.30 (0.16; 0.45)**	CG

Figure S27 Network map of ASM

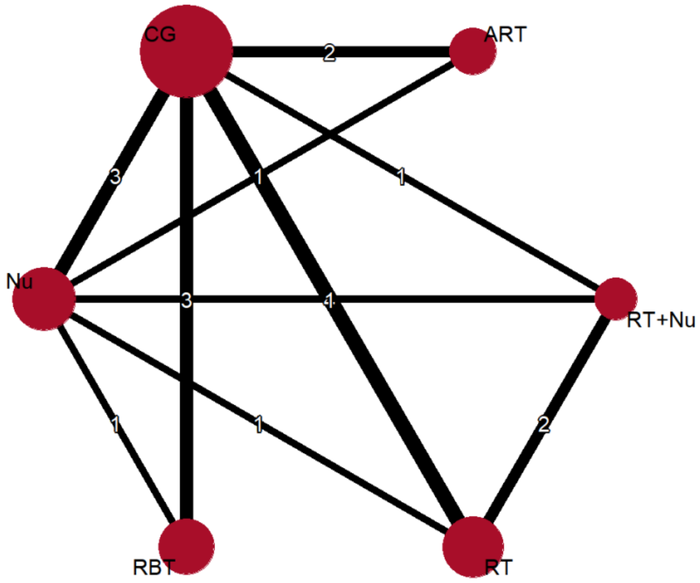

Figure S28 Forest plot of ASM

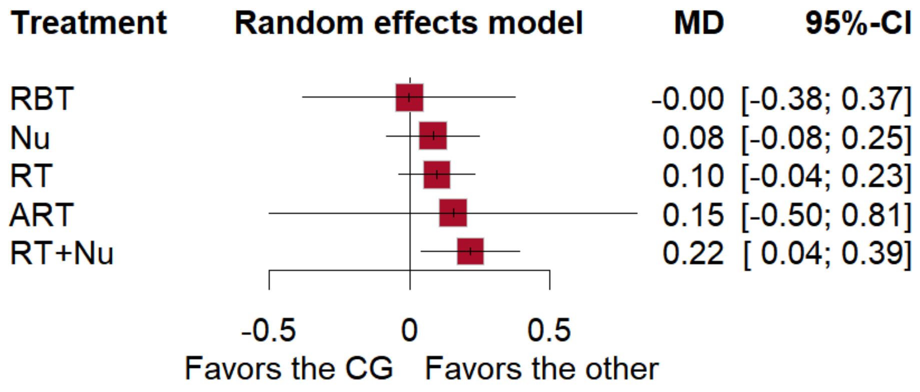

Table S29 League table of ASM

ART	0.10 (-0.99; 1.19)	.	.	.	0.13 (-0.54; 0.80)
0.07 (-0.60; 0.74)	Nu	-0.12 (-0.64; 0.40)	0.00 (-0.18; 0.18)	-0.10 (-0.31; 0.11)	0.09 (-0.08; 0.26)
0.16 (-0.60; 0.91)	0.09 (-0.30; 0.47)	RBT	.	.	-0.04 (-0.45; 0.36)
0.06 (-0.61; 0.72)	-0.01 (-0.19; 0.16)	-0.10 (-0.49; 0.29)	RT	-0.12 (-0.30; 0.05)	0.11 (-0.03; 0.25)
-0.06 (-0.74; 0.61)	-0.13 (-0.34; 0.07)	-0.22 (-0.63; 0.19)	-0.12 (-0.29; 0.05)	RT+Nu	**0.20 (0.02; 0.38)**
0.15 (-0.50; 0.81)	0.08 (-0.08; 0.25)	-0.00 (-0.38; 0.37)	0.10 (-0.04; 0.23)	**0.22 (0.04; 0.39)**	CG

Figure S29 Network map of SMI

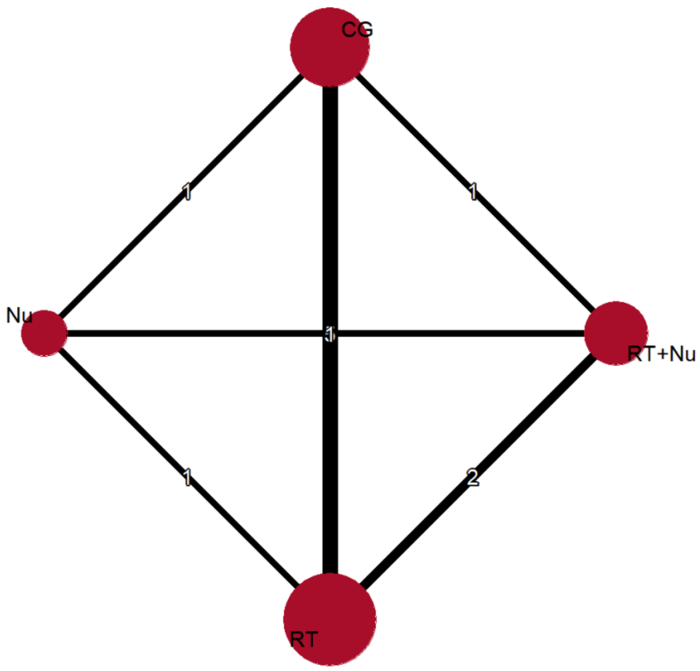

Figure S30 Forest plot of SMI

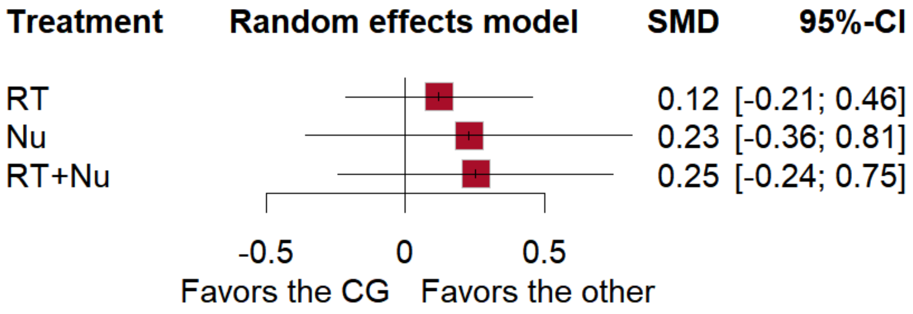

Table S30 League table of SMI

Nu	0.01<br>(-0.32; 0.34)	0.03<br>(-0.30; 0.36)	0.01<br>(-0.32; 0.34)
0.01<br>(-0.29; 0.31)	RT	-0.08<br>(-0.33; 0.16)	0.12<br>(-0.16; 0.40)
-0.04<br>(-0.34; 0.27)	-0.05<br>(-0.28; 0.19)	RT+Nu	-0.02<br>(-0.35; 0.31)
0.08<br>(-0.24; 0.39)	0.07<br>(-0.20; 0.33)	0.11<br>(-0.18; 0.40)	CG

Section S9: Sensitivity analysis

Section S9.1 Sensitivity Analysis Excluding Studies at High Risk of Bias

Figure S31 Forest plot of Handgrip strength

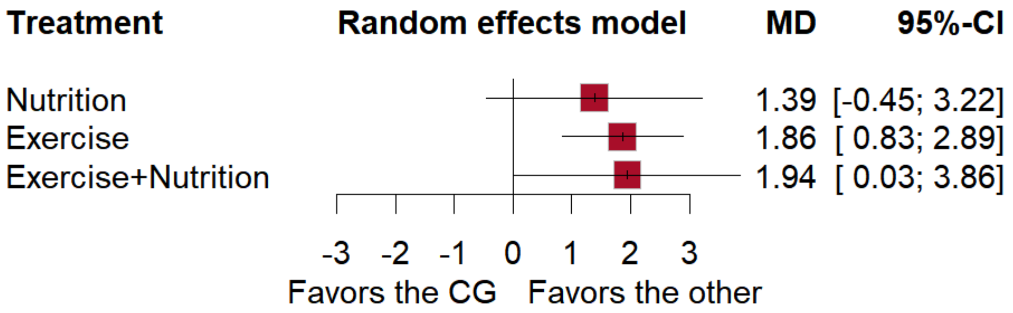

Section S9.2 Sensitivity Analysis Excluding Studies with Fewer Than 15

Figure S32 Forest plot of Handgrip strength

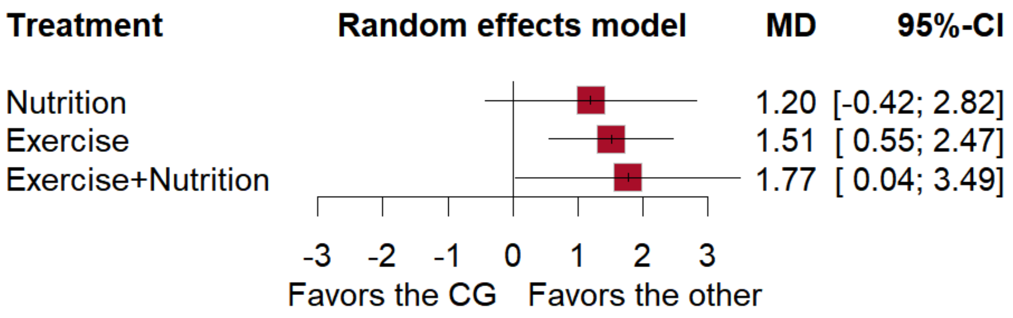

Figure S33 Forest plot of Knee extension strength

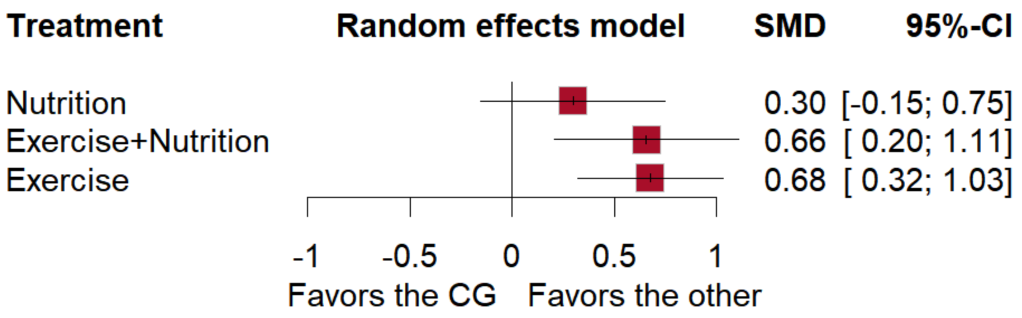

Figure S34 Forest plot of Usual gait speed

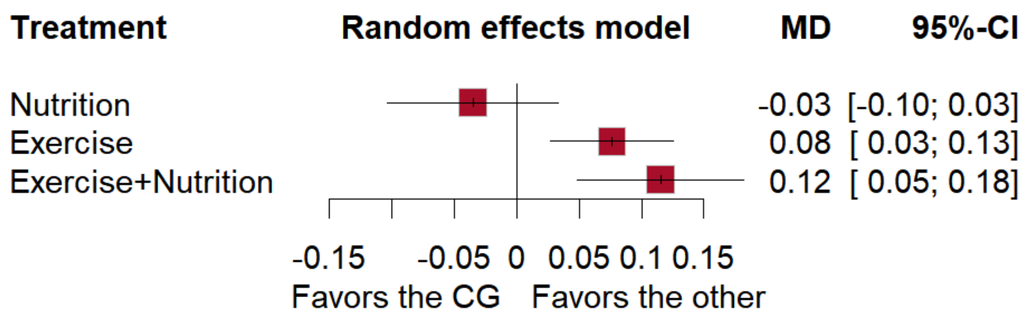

Figure S35 Forest plot of Maximal gait speed

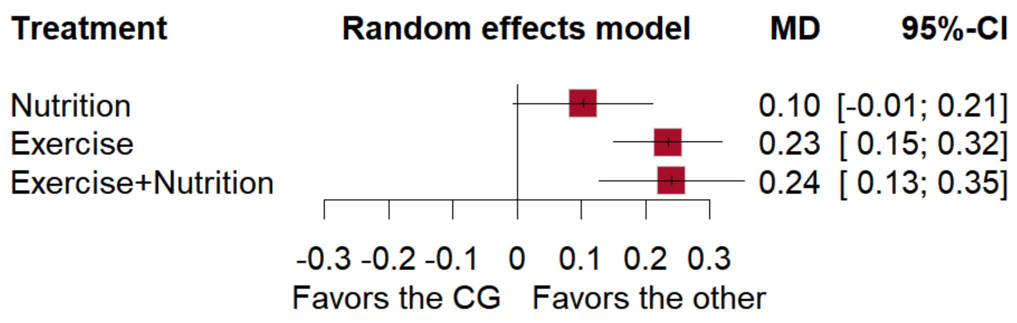

Figure S36 Forest plot of ASM

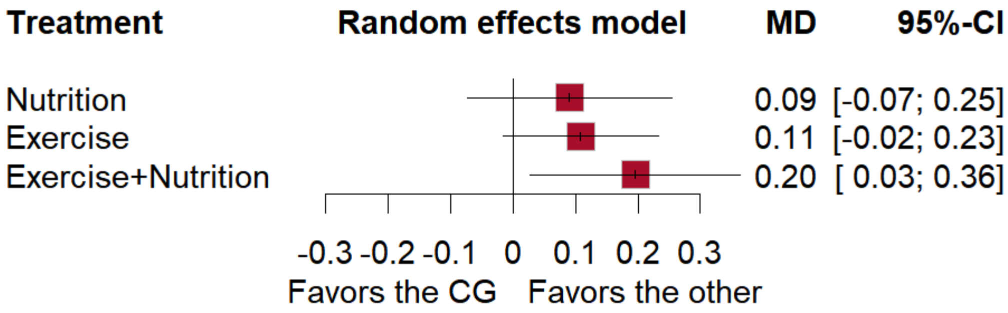

Section 9.3 Sensitivity Analysis Excluding Studies Including Participants with Probable Sarcopenia

Figure S37 Forest plot of Usual gait speed

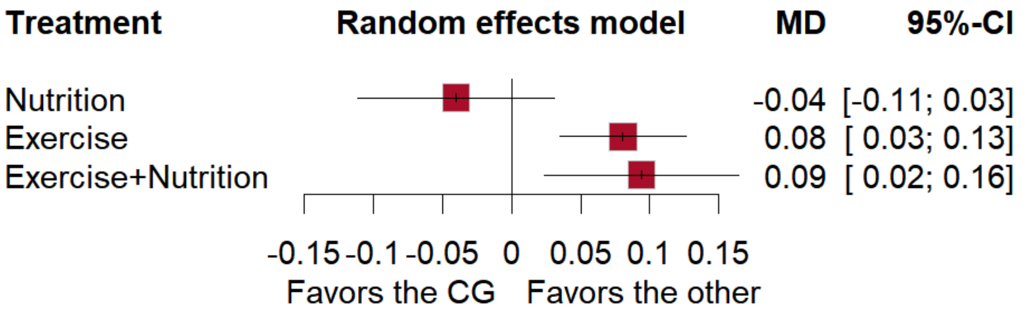

Figure S38 Forest plot of SMI

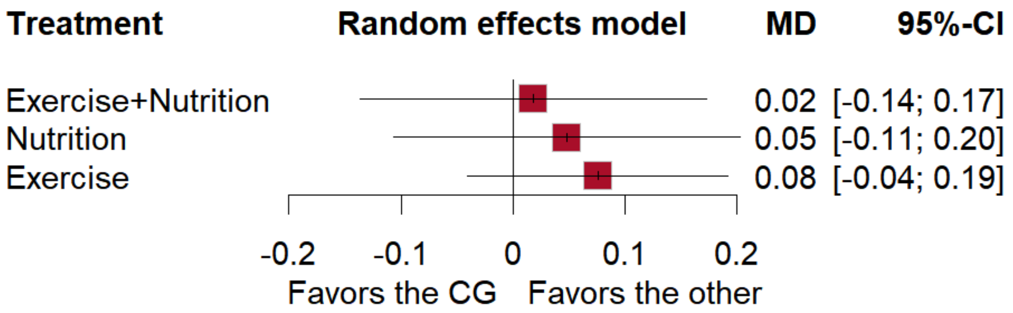

Supplement: Supplementary file 1 [file nutrients-17-02392-s001.zip › nutrients-3719148-supplementary.pdf]
